# Luminescent Behavior of Sb^3+^-Activated Luminescent Metal Halide

**DOI:** 10.3390/nano13212867

**Published:** 2023-10-29

**Authors:** Tao Huang, Bingsuo Zou

**Affiliations:** 1 State Key Laboratory of Featured Metal Materials and Life-Cycle Safety for Composite Structures, School of Resources, Environmental and Materials, Guangxi University, Nanning 530004, China; htao1201@163.com; 2 State Key Laboratory of Luminescent Materials and Devices, School of Materials Science and Engineering, South China University of Technology, Guangzhou 510640, China

**Keywords:** Sb^3+^-activated, metal halide perovskites, tunable emission, self-trapped exciton

## Abstract

Metal halide perovskites have unparalleled optoelectronic properties and broad application potential and are expected to become the next epoch-making optoelectronic semiconductors. Although remarkable achievements have been achieved with lead halide perovskites, the toxicity of lead inhibits the development of such materials. Recently, Sb^3+^-activated luminescent metal halide perovskite materials with low toxicity, high efficiency, broadband, large Stokes shift, and emission wavelengths covering the entire visible and near-infrared regions have been considered one of the most likely luminescent materials to replace lead halide perovskites. This review reviews the synthesis, luminescence mechanism, structure, and luminescence properties of the compounds. The basic luminescence properties of Sb^3+^-activated luminescent metal halide perovskites and their applications in WLED, electroluminescence LED, temperature sensing, optical anti-counterfeiting, and X-ray scintillators are introduced. Finally, the development prospects and challenges of Sb^3+^-activated luminescent metal halide perovskites are discussed.

## 1. Introduction

Metal halide perovskites (MHPs) have attracted wide attention due to their excellent optoelectronic properties and solution processability [1,2,3]. The general formula for MHPs is ABX_3_, where A is a monovalent organic or inorganic cation (e.g., Cs^+^, Rb^+^, or formamidinium (FA^+^)), B is a divalent metal cation (e.g., Pb^2+^, Cd^2+^, or Sn^2+^), and X is a halide anion (Cl^−^, Br^−^, I^−^) [4]. The history of MHPs dates back to 1893 [5], but it was not until 2009 that researchers became interested in them. Miyasaka et al. successfully applied MHPs to optoelectronic devices for the first time [6]. MHPs can self-assemble univalent cations of different properties and inorganic metal halide anions to form a new material, which can obtain a variety of derivatives by replacing organic molecules, ions, and ionic groups. Interestingly, MHPs are good at separating charge to generate electricity and aggregating charge to emit light [7,8,9,10]. In 2014, Tan et al. demonstrated for the first time at room temperature high-luminance LEDs based on solution-treated organometallic halide perovskites [11]. MHPs have made great progress in light-emitting diodes, solar cells, photodetectors, lasers, displays, and other fields over the past decade [12,13,14,15,16,17,18]. This is unmatched by other materials currently available for commercial optoelectronic devices, such as III-V semiconductors, organic materials, and conventional quantum dots (CdSe, CdS, etc.) [19,20]. MHPs have become an independent research field.

The most high-profile previous MHPs have relied on toxic lead, such as CsPbX_3_ [21,22,23], (C_9_H_22_N_2_)PbBr_4_ [24], and CH_3_NH_3_PbI_3_ [25]. This kind of MHP has great potential in light-emitting devices due to its tunable luminescence color [25,26], high photoluminescence quantum yield [27], and simple processing technology [28,29]. However, the toxicity of lead is restricted by consumer electronics regulations [30]. In addition, the instability of Pb^2+^ in light, humidity, heat, and other environments also hinders the further commercial application of MHPs [31,32]. Therefore, the development of efficient, stable, and non-toxic MHP is an urgent goal in this field. It is possible to replace the lead element with other non-toxic metal ions [33]. Previously, it was confirmed that the ^6^s_2_p_0_ electronic configuration of Pb^2+^ plays an important role in the excellent photoelectric properties of lead MHPs [34,35]. In general, metal ions with an ns^2^ electron configuration have broad emission bands and large Stokes shifts, which can effectively avoid self-absorption and are ideal luminescent ions [36,37]. Therefore, metal ions with ns^2^ electronic configurations such as Sb^3+^, Te^4+^, Bi^3+^, and Sn^2+^, which have similar electronic structures to Pb^2+^, have been proven to be a viable alternative. For example, Cs_3_Sb_2_I_9_ [38], Cs_2_TiCl_6_ [39,40], Cs_2_AgBiBr_6_ [41,42,43,44], and Cs_2_SnX_4_ [45,46]. Sb^3+^-activated luminescent MHPs (Sb-MHPs) have been widely explored due to their emission bandwidth covering the whole visible light region, low toxicity, high PLQY, and high stability [47,48,49]. The unique singlet and triplet exciton emission mechanism can realize tunable emission in a large range of visible-near infrared [50,51,52,53], and this excellent optical performance is not possessed by most other metal ions. Currently, Sb^3+^ is considered to be one of the most likely materials to replace lead halides. For example, Cs_3_Sb_2_X_9_ (X = Cl, Br, and I) is formed by the substitution of Sb^3+^ for 2/3 of the sites occupied in CsPbX_3_. Another typical Sb^3+^-based metal halide, Cs_2_AgSbCl_6_, has a similar cubic structure to CsPbX_3_ [37]. Their unique singlet and triplet exciton emissions have been discussed in many studies using a combination of experimental and theoretical methods.

In this review, the synthesis methods and luminescence properties of Sb^3+^-activated luminescent MHPs (Sb-MHPs) in recent years are reviewed, as well as the optical properties and luminescence mechanisms of different types and different emission colors of Sb^3+^-activated luminescent MHPs. Then, we discuss the latest situation and advantages of Sb^3+^-activated luminescent MHPs as emission layers in practical applications, such as the unique singlet and triplet emission of Sb^3+^ in a white light-emitting diode (WLED), anti-counterfeiting, temperature sensing, and other fields. Finally, we offer some suggestions on the challenges faced by Sb-MHPs and discuss their development prospects.

## 2. Synthesis

Since Miyasaka et al. prepared optoelectronic devices in 2009 [6], innovative breakthroughs have been made in MHP synthesis methods. Previously, most of the reported MHPs were prepared by the liquid growth method [54,55,56,57], and a few materials were prepared by vapor deposition [58,59]. This paper mainly discusses the most widely used liquid growth method in the preparation of Sb-MHPs. For example, the hot injection method and the ligand-assisted reprecipitation (LARP) method are used to prepare quantum dots (QDs) and nanocrystals (NCs). The crystal was prepared by the anti-solvent method, the hydrothermal method, and the solvent slow evaporation method. A thin film was prepared by the spinning coating method.

### 2.1. Quantum Dots (QDs) and Nanocrystals (NCs)

**Figure 1:**
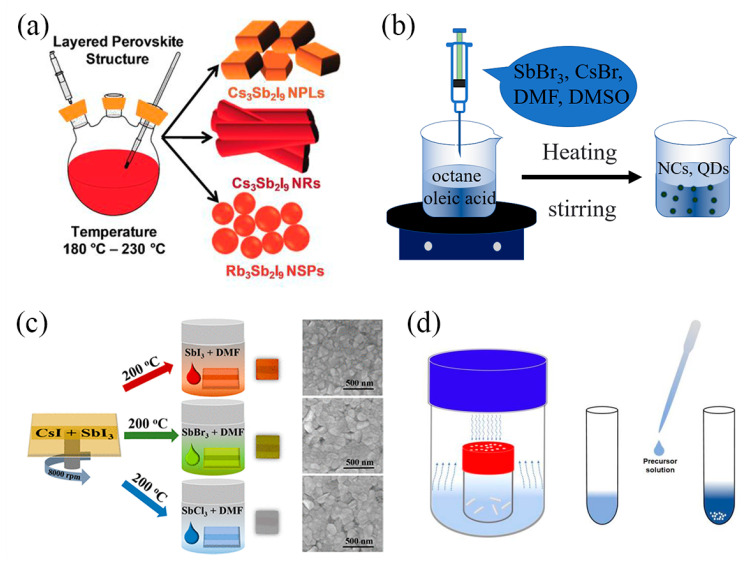
(**a**) Synthesis of A_3_SbI_9_ (A = Cs, Rb) NCs by hot injection. Reprinted with permission from Ref. [38]. Copyright 2020 Wiley. (**b**) Synthesis of Cs_3_SbX_9_ NCs or QDs by thermal injection. Adapted with permission from Ref. [31]. Copyright 2017 American Chemical Society. (**c**) Synthesis of Cs_3_Sb_2_X_9_ thin films by spinning coating. Reprinted with permission from Ref. [69]. Copyright 2019 American Chemical Society. (**d**) Synthesis of (TMA)_2_SbCl_5_ crystals by antisolvent method. Reprinted with permission from Ref. [48]. Copyright 2021 American Chemical Society.

The most common method for the synthesis of Sb-MHPs, QDs, or NCs is the hot injection method. The hot injection method was first proposed by Murray et al. in 1993 for the synthesis of CdS QDs. In 2015, Protesescu et al. synthesized inorganic lead cesium halide perovskite QDs by the hot injection method for the first time [60]. In this method, the precursor solution is rapidly injected into the reaction solvent with a high temperature and boiling point, immediately forming nanocrystals [61,62]. When nanocrystals are synthesized by this method, the morphology, size, stability, and optical properties of nanocrystals can be controlled by changing the reaction temperature, crystal growth time, and ligand type [18,63,64]. In 2017, Pal et al. synthesized two Cs_3_Sb_2_I_9_ NCs with different morphologies by hot injection [38], as shown in Figure 1a. They placed SbI_3_ and ODE (1-Octadecene) in a three-necked round-bottom flask, degassed, and stirred at 80 °C for 60 min. OnA (octanoic acid) and OAm (Oleylamine) were added to the mixture at 80 °C under an N_2_ atmosphere. Then the reaction temperature was raised to 140 °C to dissolve SbI_3_. Afterward, pre-synthesized Cs-oleate was dissolved in the ODE at 100 °C and then rapidly injected into the reaction mixture at 180 °C and 230 °C. Within a few seconds, the reaction mixture turned cloudy red, and after 1 min, the reaction flask was immersed in an ice bath to obtain Cs_3_Sb_2_I_9_ NCs. By changing the reaction temperature, they can adjust the morphology of Cs_3_Sb_2_I_9_ NCs nanoplates (NPLs) and nanorods (NRs). They also obtained Rb_3_Sb_2_I_9_ nanocrystals using a similar method. In 2021, Zhang et al. also used hot injection to obtain Rb_7_Sb_3_Cl_16_ and Sb-doped Rb_3_InCl_6_ NCs [65]. In 2020, Cai et al. synthesized Cs_4_CuSb_2_Cl_12_ double perovskite-type NCs by hot injection for the first time [66]. By gradually adjusting the material composition and feed ratio (Cs_4_Cu_x_Ag_2−2x_Sb_2_Cl_12_, 0 ≤ x ≤ 1), the crystal structure and the corresponding electronic band structure band gap are realized.

Ligand-assisted reprecipitation (LARP) is also a common method used to prepare Sb-MHP QDs or NCs [31]. The simple process is to supersaturate and recrystallize the solution by changing the reaction conditions, but it requires certain ligands to precipitate the crystal, which can control the formation and growth of the crystal to the nanometer scale. The LARP method can be performed in the air using a simple device, as shown in Figure 1b. In 2017, Zhang et al. prepared Cs_3_Sb_2_Br_9_ colloidal quantum dots by the LARP method [31]. They dissolved 0.3 mmol CsBr and 0.2 mmol SbBr_3_ in 2 mL of DMF or DMSO, and then 10 μL of oleylamine or n-octylamine was added to the mixture to form a precursor solution. 1 mL of the precursor solution was vigorously stirred and quickly dropped into a mixture of 10 mL octane and 2 mL OA. After a few seconds of reaction, a strong blue emission of quantum dots can be observed. In 2019, Lv et al. synthesized Sb-based Cs_2_AgSbX_6_ (X = Cl, Br, or I) double perovskite quantum dots for the first time using LARP technology [67]. They rapidly dropped a mixture of DMSO, CsX, AgX, and SbX_3_ into a mixture of oleic acid (OA) and ethyl acetate at room temperature, resulting in Cs_2_AgSbX_6_ quantum dots within seconds. The material has good air stability and strong blue emission, and the photoluminescence quantum yield is 31.33%. In 2022, Yang et al. synthesized highly efficient near-UV luminescence colloidal Cs_2_NaBi_0.75_Sb_0.25_Cl_6_ nanocrystals with a PLQY of 39.5% using LARP technology [68]. Different from the hot injection method, the synthesis temperature of this method is low, the reaction time is short, and it does not need inert gas protection. And it can easily mass-produce MHP NCs. However, it is difficult to obtain uniform-size products by this method.

### 2.2. Films and Single Crystals

The spin-coating method is commonly used to prepare Sb-MHP films by spinning the prepared precursor onto a clean substrate. In 2019, Singh et al. prepared the precursor solution by mixing SbI_3_, CsI, and DMSO and then stirring continuously for 6 h at 70 °C [69]. The solution was dropped onto a substrate coated with poly (ethylene dioxythiophene): polystyrene sulfonate (PEDOT: PSS), and the coating was then spun at 8000 rpm for 40 s to yield Cs_3_Sb_2_I_9_ films. The films were preheated at 200 °C and annealed at 200 °C for 15 min, as shown in Figure 1c. Buonassisi et al. prepared three compounds, A_3_Sb_2_I_9_, using a two-part spin coating method (where A is Cs, Rb, and K) [70]. Firstly, when AI and SbI_3_ are dissolved in a mixed solution of DMF and DMFO, the molar ratio of AI and SbI_3_ is fixed at 3:2. Sediment the Sb-based solution onto the pre-cleaned substrate through a two-step spin coating procedure (first step: 1000 rpm, 10 s; second step: 5000 rpm, 20 s). In the second spin coating step, chlorobenzene drops onto the rotating substrate within~5 s, and then the thin film is sintered on a hot plate in the glove box at 100 °C for 20 min.

With the rapid development of Sb-MHP single crystal preparation technology, anti-solvent methods, hydrothermal methods, solvent slow evaporation, and other methods have been widely used in the synthesis of various high-quality Sb-MHP single crystals. In the anti-solvent method, reactants are dissolved in a solvent to form precursors, and then an anti-solvent is added. Due to the combination of anti-solvent and solvent, the solubility of products to be produced in the solvent is reduced, and a supersaturated solution is produced, thus precipitating crystal products. In 2021, Wei et al. synthesized the (TMA)_2_SbCl_5_·DMF single crystal by the antisolvent method [48], as shown in Figure 1d. First, 0.5 mmol TMA·HCl and 0.25 mmol SbCl_3_ (molar ratio 2:1) were dissolved in 2 mL and 1 mL DMF, respectively, and then mixed in a vial. Ethyl ether was placed in large vials and slowly diffused into 1 mL of mixed DMF solution at room temperature. After some time, colorless bulk crystals were obtained. In 2022, Peng et al. synthesized the (C_16_H_28_N)_2_SbCl_5_ single crystal by a similar method [71]. They completely dissolved SbCl_3_ (0.2281 g) and C_16_H_28_NCl (0.5397 g) in DMF at room temperature (RT) to form a transparent solution. (C_16_H_28_N)_2_SbCl_5_ SCs were obtained by diffusing them into a DMF solution with diethyl ether at room temperature and then overnight. The products prepared by the anti-solvent method have high purity and few impurities, but more toxic solvents are used. In the hydrothermal method, reactants and solvents are added to the reaction kettle together, and the reactant raw materials can be completely dissolved under high temperature and pressure for some time. Slow cooling causes the solution to reach a highly supersaturated state and precipitate crystal nuclei, which begin to grow. The most important characteristics of the hydrothermal method are the high purity of products, fewer impurities, controllable crystal size, and low production cost. For example, Huang et al. synthesized a series of Sb-doped (Cs_1−x_Rb_x_)_2_InCl_5_∙H_2_O and (Cs_1−x_Rb_x_)_3_InCl_6_ halide perovskites by hydrothermal method using concentrated hydrochloric acid (HCl) and methanol (MeOH) as reaction solvents [47]. They obtained products with different emission colors by changing the Cs/Rb feed ratio. In 2022, Chang et al. prepared a series of Sb-doped CD-based perovskites (Cs_7_Cd_3_Br_13_, Cs_2_CdCl_2_Br_2_, and Cs_3_Cd_2_Cl_7_) with different phases by the hydrothermal method and effectively modulated PL emission by halogen substitution.

## 3. Optical Properties

### 3.1. Mechanism of Luminescence of Sb^3+^

**Figure 2:**
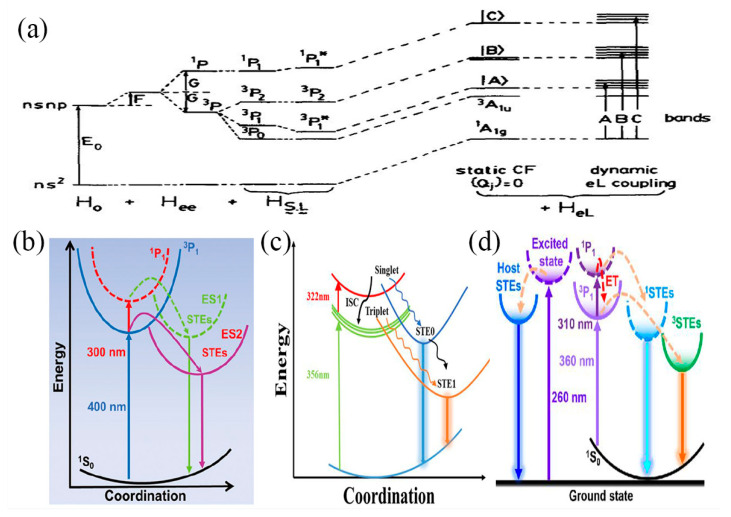
(**a**) Energy level diagram for an ion with the ns^2^ ground-state configuration. Reprinted with permission from Ref. [72]. Copyright 1991, Elsevier. (**b**) Coordinate diagram of a photophysical process of (C_16_H_28_N)_2_SbCl_5_. Reprinted with permission from Ref. [71]. Copyright 2022, Springer. (**c**) Coordinate diagram of a photophysical process of Sb^3+^: RbCdCl_3_. Reprinted with permission from Ref. [79]. Copyright 2022, American Chemical Society. (**d**) Coordinate diagram of a photophysical process of Sb^3+^: Cs_2_ZrCl_6_. Reprinted with permission from Ref. [80]. Copyright 2022, American Chemical Society.

The electronic configuration of the Sb^3+^ ion is 5s^2^, corresponding to the electronic ground state ^1^S_0_, and the first excited state is nsnp, which gives rise to the four energy states of ^1^P_1_, ^3^P_0_, ^3^P_1,_ and ^3^P_2_ in the Russell–Saunders (RS) coupling, as shown in Figure 2a [72]. In this figure, E_0_ represents the energy difference between the ground state and the excited state, H_0_ contains the electron–nucleus interaction and Madelung potential, F is the electron-electron interaction resulting in Coulomb repulsion, and G is the exchange interaction. However, the RS coupling is an inappropriate approximation much larger than n, so the electron interaction (H_ee_) + spin-orbit coupling (H_S·L_) matrix is needed to find the energy level. The diagonal term in the spin-orbit coupling H_S·L_ (Russel–Saunders coupling) decompositions the ^3^P term into three components with J = 0, 1, 2, defines ^3^P_2_, ^3^P_1,_ and ^3^P_0_ as triplet states, and defines ^1^P_1_ as a singlet state [73]. The splitting of the energy levels is caused by the electron interaction and the spin-orbit coupling matrix, * indicates that ^3^P_1_*, and ^1^P_1_* are not pure RS states but are formed from a mixture of off-diagonal matrix elements. The spin–orbit interaction separates the degenerate ^3^P level but also mixes the ^3^P_1_ and ^1^P_1_ states. According to Hund’s rules, the energies of these excited states from ^3^P_0_ < ^3^P_1_ < ^3^P_2_ < ^1^P_1_ start to increase. As a result, the transitions of ^1^S_0_–^1^P_1_ are parity-allowed, and the transitions of ^1^S_0_–^3^P_1_ are spin-forbidden, but spin–orbit coupling relaxes this selection rule and is also parity-allowed (relaxation in the states of ^3^P_1_ and ^1^P_1_). The ^1^S_0_–^3^P_1_ transition is a lower-energy transition. The transition of ^1^S_0_–^3^P_0_ or ^1^S_0_–^3^P_2_ is strictly forbidden.

Under photoexcitation, the Sb^3+^ electrons move from the ground state ^1^S_0_ to the excited states ^1^P_1_ (triplet) and ^3^P_1_ (triplet). ^3^P_1_ and ^1^P_1_ can be coupled to different multiple phonons, resulting in singlet and triplet STEs [74,75,76]. The energy is transferred between singlet STEs and triplet STEs by intersystem crossing (ISC), which is generated by spin-orbit coupling. In general, S–T transformations have a temperature dependence, which is consistent with spin–orbit interactions in lattices, so singlet transitions are usually of low strength at room temperature (RT) [77,78]. Peng et al. proposed that (C_16_H_28_N)_2_SbCl_5_ is excited from the ^1^S_0_ excited state to the excited state (^3^P_1_ and ^1^P_1_) under UV irradiation (such as 300 nm) [71]. Subsequently, excited electrons are rapidly transferred to triplet states via ISC and then returned to GS (ground state) to achieve radiative recombination of singlet and triplet STEs, as shown in Figure 2b. The transfer of power between singlet (S) and triplet (T) states also depends on temperature. Meng et al. proposed that excitation of Sb^3+^:RbCdCl_3_ at 322 nm could produce singlet emission and triplet emission of Sb^3+^, and then presented singlet emission bands belonging to the ^1^P_1_–^1^S_0_ transition at low temperature (460 nm) and triplet emission bands belonging to ^3^P_n_–^1^S_0_ at room temperature (596 nm) [79], as shown in Figure 2c. This fact reflects that the S-T transformation is strongly temperature-dependent. In addition, singlet and triplet excitons can exhibit two adjustable emission peaks at different excitation wavelengths. For example, Zhang et al. proposed the photoexcited radiation process of Cs_2_ZrCl_6_:XSb [80], as shown in Figure 2d. At 310 nm, the ground-state electrons are excited to the high-energy excited state, and the transitions of ^1^S_0_ → ^1^P_1_ and ^1^S_0_ → ^3^P_1_ occur, accompanied by an energy transfer from ^1^P_1_ to ^3^P_1_. At the same time, the electrons in the high-energy excited state can be captured by ^1^STE and ^3^STE. Eventually, electrons recombine from ^1^STE and ^3^STE to the ground state, resulting in two-band emission with a large Stokes shift. At low-energy photoexcitation (360 nm), the ^3^STEs exciton emission becomes stronger.

### 3.2. Structure and Luminescence of Sb-MHPs

#### 3.2.1. Dimensions in Size

**Figure 3:**
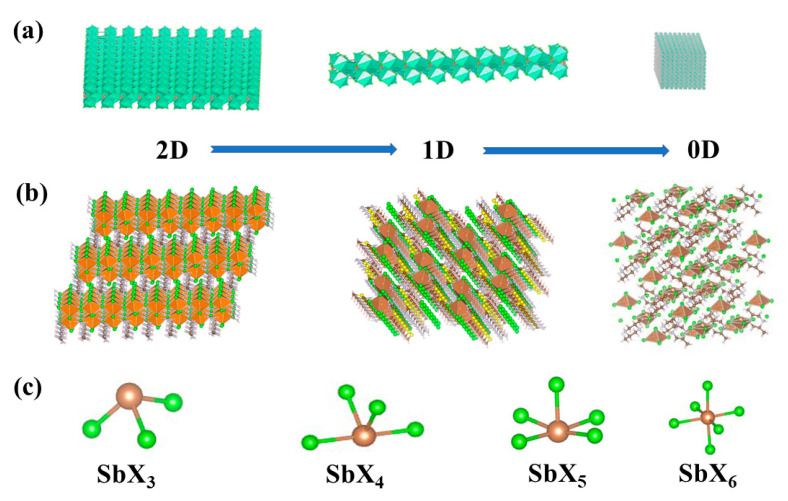
(**a**) Morphological dimension reduction of Sb-MHPs. (**b**) Molecular dimension reduction of Sb-MHPs. (**c**) The coordination environment of Sb^3+^ in Sb-MHPs.

In addition to 3D perovskite, the Sb-MHPs system is consistent with other perovskite systems, and 0D structured quantum dots [65], 1D structured nanowires [38], and 2D structured nanosheets [81] can be prepared by a dimensional reduction in size, as shown in Figure 3a. Essentially, they are still composed of 3D metal halide frameworks; their properties are influenced by 3D perovskites and the quantum confinement effect; and they typically exhibit small Stokes shifts, narrow emission, and the quantum confinement effect [31]. Sb-MHP materials with different dimensions have excellent performance in optoelectronic properties and are often used to prepare various optoelectronic devices.

#### 3.2.2. Dimensions at the Molecular Level

In the three-dimensional Sb-MHPs structure, when the cations located at the A site are replaced by large-sized organic cations, the complete three-dimensional perovskite structure will not be able to accommodate the large-sized organic cations due to the voids between the metal halide frameworks [82]. They are separated by layers of organic cations to form low-dimensional structures at the molecular level (isolated layer, isolated line, or isolated polyhedron), as shown in Figure 3b. In this case, the individual metal halide layers, long chains, or polyhedra inside the bulk crystal are isolated from each other by organic cations, enabling the bulk crystal to display the intrinsic properties of individual building blocks as well as quantum confinement effects at the molecular level. The transformation of 3D Sb-MHPs to lower dimensions allows great flexibility in the structure. The 2D Sb-MHPs are subject to quantum confinement and dielectric confinement effects in a specific direction, with a natural quantum well structure, large exciton binding energy (Eb), and excellent thermal stability [83]. The 1D Sb-MHPs behave at the molecular level as single, long chains separated by organic cations. The exciton-phonon interaction in the crystal is very strong, and the excitons are more likely to be self-trapped at this time, and they usually have large Stokes displacements [84]. The metal halide polyhedrons of 0D Sb-MHPs are separated by organic molecules, are independent of each other, exist alone, and form independent luminescent centers. It preserves the photophysical properties of individual metal halide polyhedra (e.g., strong Stokes shift, broadband emission, high PLQY, and long lifetime) [48]. Furthermore, a large number of broadband emissions with large Stokes shifts are observed in low-dimensional Sb-MHPs [50,85,86], generally believed to originate from excited-state STEs due to enhanced electron-phonon coupling. Electron-phonon coupling generally refers to the interaction between electrons and lattice vibrations, which significantly affects the electrical and optical properties of semiconductors. This is because organic cations have strong vibrational modes, and the incorporation of organic molecules can soften the lattice and generate deformation potential and local lattice distortion, which greatly enhances the electron-phonon coupling of the system. Therefore, in low-dimensional Sb-MHPs, the strength of electron-phonon coupling and the formation of STEs play crucial roles.

#### 3.2.3. Coordination of Sb^3+^

In Sb-MHPs, the lone electron pairs of Sb^3+^ have a strong stereochemical effect on the coordination environment [87], and antimony halides can form coordination structures based on pyramidal MX_3_ units [38], seesaw MX_4_ units [88], square pyramid MX_5_ units [48], and octahedral MX_6_ units [88], as shown in Figure 3c. These coordination structures can exist in isolated units or in long-chain or planar forms through shared halogen atoms. Others exist in the form of polyaggregates. For example, M_2_X_8_, M_2_X_10_, M_2_X_12_, and M_3_X_12_ [89]. How these coordination structures affect the luminescence of Sb-MHPs is unclear, but the lone electron pairs of Sb^3+^ distort these coordination structures, resulting in lattice distortions that enhance the emission of STEs.

### 3.3. Sb^3+^-Doped and Un-Doped Luminescent Behaviors

#### 3.3.1. Sb^3+^-Based

Fully inorganic Sb^3+^-based metal halides are usually present as A_3_Sb_2_X_9_ (A = Cs, Rb; X = Cl, Br, I) crystals, which are formed by substitution of Sb^3+^ for sites in CsPbX_3_ crystals. Cs_3_Sb_2_I_9_ single crystals reported by Kyle M. McCall et al. have a broad asymmetric emission band at 560–770 nm, which is more typical of triplet-state STE emission [90]. In addition, the Rb_7_Sb_3_Cl_16_ crystal powder was reported by Tang et al. [91]. The Rb_7_Sb_3_Cl_16_ powder they synthesized has a broad STE yellow emission band at 560 nm with a PLQY value of 26%. For widely reported organic-inorganic hybrid Sb^3+^-based halide crystals, the luminescence is usually dominated by single- and triplet-state STE emission. Sb in Sb^3+^-based organic-inorganic hybrid metal halides usually exists in the form of Sb-X polyhedral clusters, which are separated by large organic molecules and form 0D structures at the molecular level. For example, we recently reported that (BTMAC)_2_SbCl_5_ has dual emission bands: the longer-lived triplet STE emission band (619 nm) and the shorter-lived singlet STE emission band (470 nm) [92]. Usually only the characteristic STE emission bands of Sb^3+^, i.e., triplet and singlet STE emission, are present in Sb^3+^-based metal halide crystals.

Currently, studies of Sb^3+^-based metal halide quantum dots usually focus on fully inorganic A_3_Sb_2_X_9_ NCs. Such quantum dots usually exhibit narrow-band emission with band edges. Angshuman et al. reported Cs_3_Sb_2_I_9_ nanorods and nanosheets with band-edge emission with half-peak widths of 32 nm and 30 nm, respectively [38]. Shan et al. realized an electrically driven violet LED at 408 nm by using Cs_3_Sb_2_Br_9_ quantum dots as light emitters [93]. In addition, they synthesized Cs_3_Sb_2_X_9_ quantum dots with different band gaps by adding different halogen ions, and their band-edge emission can be tuned in the range of 385~640 nm. This narrow band-edge emission of the quantum dots is clearly different from the STE broadband emission of the bulk.

#### 3.3.2. Sb^3+^-Doped

In our previous work, we found that Sb^3+^ as a doping agent can introduce its characteristic STE emission into many metal halide crystals. We have observed STE emissions from Sb^3+^ in systems such as Sb^3+^-Doped RbCdCl_3_ [94], Sb^3+^-Doped Cs_3_InCl_6_ [47], and Sb^3+^-Doped (C_13_H_30_N)_2_SnCl_6_ [95]. Compared to Sb^3+^-based halides, the STE emission efficiency generated by Sb^3+^ as a doping agent is particularly high. In addition, in some systems, doping Sb^3+^ can still retain the emission band of the host ion itself and tune different emission centers. For example, Li et al. synthesized a (BTPP) _2_MnCl_4_:Sb with STE emission of Sb^3+^ and ^4^T_1_–^6^A_1_ transition of Mn^2+^ ions [96]. By changing the excitation wavelength, the emission of green, yellow, and orange can be adjusted. Li et al. also found a similar phenomenon in (TTPhP)_2_MnCl_4_:Sb^3+^ [97]. At the same time, they also found that STE emissions from Sb^3+^ and Mn^2+^ exhibit different thermal quenching effects, leading to the excellent temperature sensitivity of this material. As mentioned above, Sb^3+^ as a dopant can be introduced into multiple emission centers, enabling efficient, tunable emission.

Unlike the band-edge narrow-band emission of A_3_Sb_2_X_9_ NCs, the emission of Sb^3+^ as a dopant in nanocrystals is usually derived from the broadband emission of STEs. Xu et al. observed triplet-state STE emission from Sb^3+^ in Sb-doped Cs_2_NaInCl_6_ NCs, showing blue emission with 84% high PLQY [26]. Hens et al. observed strong cyan emission in Cs_2_CdCl_4_:Sb^3+^ nanoplatelets, which also comes from the triplet state of Sb^3+^ [81].

### 3.4. Different Colors of Emission

#### 3.4.1. Ultraviolet (UV) Emission and Blue Emission

**Figure 4:**
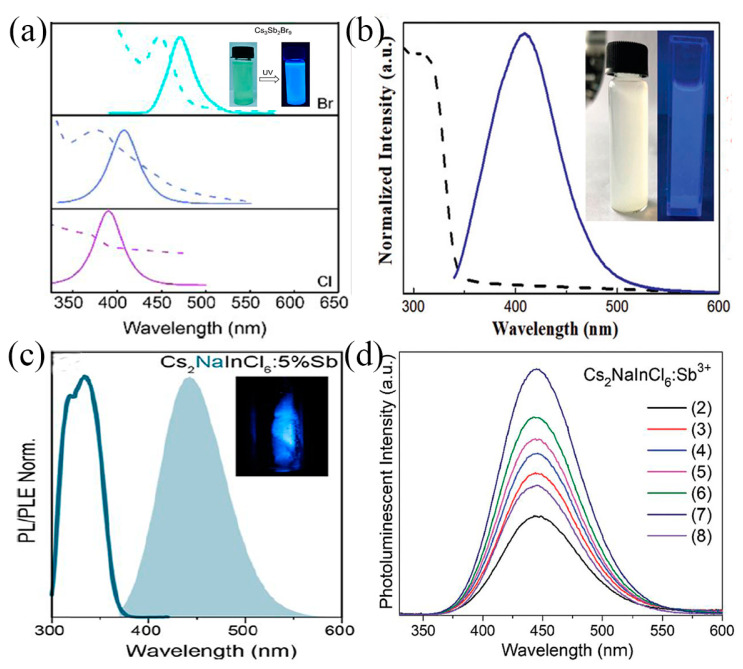
(**a**) Composition-tunable absorption and PL spectra of Cs_3_Sb_2_X_9_ (X = Cl, Br) IPQDs by halide substitution. Reprinted with permission from Ref. [31]. Copyright 2017, American Chemical Society. (**b**) Absorption and PL spectra of Cs_2_AgSbCl_6_ QD. Reprinted with permission from Ref. [67]. Copyright 2019, Royal Society of Chemistry. (**c**) PL (under 320 nm UV excitation) and PLE of Cs_2_NaInCl_6_:5%Sb SCs. Reprinted with permission from Ref. [99]. Copyright 2020, American Chemical Society. (**d**) Emission spectra of Cs_2_NaInCl_6_:Sb^3+^ samples (The numbers (2)–(8) represents a ratio (%) of 0.1, 0.5, 1.0, 2.5, 5, 10, 20, 50 for Sb^3+^ and In^3+^ in the solution, respectively). Reprinted with permission from Ref. [100]. Copyright 2020, Royal Society of Chemistry.

At present, blue light exists in a large number of computer monitors, fluorescent lamps, mobile phones, digital products, LEDs, and other lights and has become an essential light source for daily life. A large number of reports have proved that Sb-MHP is a promising blue emission material without rare earth. Generally, when Sb-MHP is prepared into QDs, due to the strong quantum domain-limiting effect in the system, the emission peak usually shows a strong blue shift, and its emission wavelength may also be located in the ultraviolet or blue light region. In 2017, Zhang et al. reported a series of inorganic perovskite QDs (Cs_3_Sb_2_X_9_) [31], in which Cs_3_Sb_2_Cl_9_ QDs showed UV emission with an emission peak of 370 nm. At the same time, Cs_3_Sb_2_Br_9_ QDs with blue (410 nm) emission were obtained by an anion exchange reaction, as shown in Figure 4a. Both can maintain high quantum yields (10~46%) and full width at half maximum (FWHM) of 40~60 nm. Compared with Cs_3_Sb_2_Br_9_ SCs, the QD emission peak of Cs_3_Sb_2_Br_9_ QDs showed a blue shift of 120 nm, showing a strong quantum confinement effect. This all-inorganic lead-free perovskite with high PLQY and stable emission quantum dots offers great potential for efficient emission candidates. Subsequently, Zhang et al. reported Cs_2_AgSbX_6_ QDs [67]. Figure 4b shows the absorption spectrum and photoluminescence (PL) spectrum of Cs_2_AgSbCl_6_ QDs, showing an exciton absorption peak at 325 nm and an emission peak at 409 nm. The inset shows a photograph of QDs illuminated with a 325 nm UV lamp, where bright blue emission can be observed. A blue shift of about 120 nm was similarly found compared to the previously reported bulk single crystals [98], also indicating a considerable quantum confinement effect of perovskite QDs. At the same time, they also mentioned blue-emitting Cs_2_AgSbBr_6_ QDs with an emission peak of 478 nm. Lead-free Sb-based blue emission perovskite quantum dots exhibit excellent optical properties, making them highly promising candidates for future optoelectronic applications.

Sb^3+^ in Sb-MHP transitions from the excited state ^3^P_1_ to the ground state ^1^S_0_ under photo-excitation; this low-energy transition might also be in the blue region. In 2020, Noculak et al. reported Sb-doped Cs_2_NaInCl_6_ single crystals [99], which exhibited broadband blue PL emission with an emission peak at 445 nm (Figure 4c), and the Stokes shifts were 110 nm. In particular, when doped with 5%Sb, its PLQY reaches the highest value of 79%. While the undoped Cs_2_NaInCl_6_ compound showed no PL emission at RT. This blue emission can be attributed to the ^3^P_1_–^1^S_0_ transition and belongs to the triplet emission of Sb^3+^. At the same time, Gray et al. also reported the intense blue emission of Cs_2_NaInCl_6_:Sb^3+^ SCs with 445 nm (Figure 4d) as the emission center and a PLQY of 79% [100]. They suggested that the reduced Stokes shift of Sb^3+^ triplet emission in Cs_2_NaInCl_6_ double perovskite relative to other types of perovskites might be due to the coordination number 6 of Sb^3+^ in Cs_2_NaInCl_6_. In addition, Cs_2_NaInCl_6_:Sb^3+^ exhibits excellent air and water stability, and these properties make it a promising blue phosphor for applications.

#### 3.4.2. Cyan Emission and Green Emission

**Figure 5:**
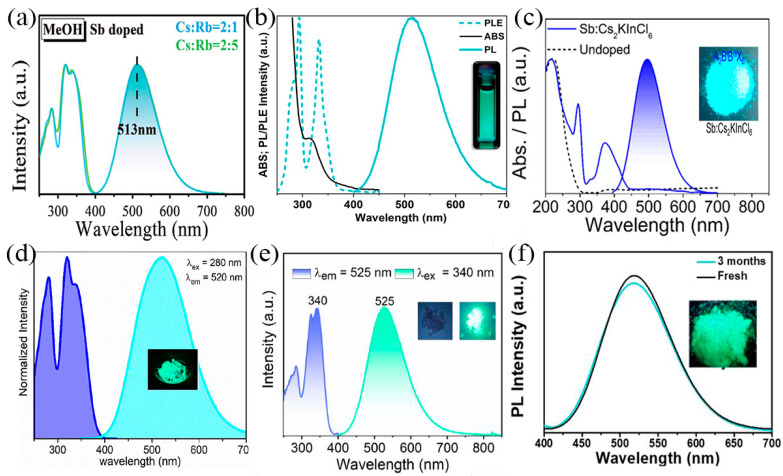
(**a**) PL and PLE spectra of Sb^3+^: (Cs_1−x_Rb_x_)_3_InCl_6_. Reprinted with permission from Ref. [47]. Copyright 2021, Wiley. (**b**) Abs, PL, and PLE spectra of Cs_2_CdCl_4_:Sb^3+^ NPs. Reprinted with permission from Ref. [81]. Copyright 2021, American Chemical Society. (**c**) Absorption spectra of undoped and Sb-doped Cs_2_KInCl_6_, and PL spectrum of 14%Sb:Cs_2_KInCl_6_. Reprinted with permission from Ref. [101]. Copyright 2022, American Chemical Society. (**d**) emission of Sb^3+^ doped Rb_3_InCl_6_. Reprinted with permission from Ref. [85]. Copyright 2020, American Chemical Society. (**e**) PL spectra of Rb_4_CdCl_6:_0.1Sb^3+^ at RT. Reprinted with permission from Ref. [77]. Copyright 2022, American Chemical Society. (**f**) PL spectra of Sb:Cs_3_Cd_2_Cl_7_ samples kept in air. Reprinted with permission from Ref. [49]. Copyright 2022, American Chemical Society.

The Sb-MHPs of Cyan emissions have also been widely reported. The source of the cyan and green emissions is the triplet emission of Sb^3+^. Previously, we found that the emission tunability of MHPs can be achieved in Sb^3+^:(Cs_1−x_Rb_x_)_3_InCl_6_ by controlling the Rb/Cs feed ratio. When the Rb/Cs feed is 2/1 or 2/5, ultra-high-efficiency cyan emission can be achieved, as shown in Figure 5a [47]. The PLQY of this highly efficient emission of cyan phosphor can be up to 90%. Alternatively, Locardi et al. also found cyan emission from the triplet of Sb^3+^ in two-dimensional Cs_2_CdCl_4_:Sb^3+^ nanoplatelets (Figure 5b) [81]. The nanoplatelets make it possible to use these compounds as optoelectronic materials. Recently, we found cyan emission from the triplet state of Sb^3+^ in Sb^3+^-doped Cs_2_KInCl_6_ double perovskite, as shown in Figure 5c [101]. Sb^3+^-doped Cs_2_KInCl_6_ double perovskite has the three-dimensional structure of a co-angular octahedron, good photoelectric performance, and high structural stability, which makes it a good candidate material for further photoelectric applications.

As one of the three primary colors of color light, green light is a sensitive color of the eyes and is widely used in lighting, transportation, medical treatment, and other fields. The Sb-MHPs of green emissions have attracted much attention due to their PLQY, low toxicity, low cost, and other advantages [94,102]. In 2020, Biswas et al. reported the green-emitting organic–inorganic hybrid Sb-based metal halide C_12_H_52_Cl_18_N_8_O_4_Sb_3_ [102]. The optical properties of this compound showed a strong and broad photoluminescence (PL) emission band centered at 517 nm with a FWHM of 110 nm. The long emission lifetime component they observed highlights that radiative emission (phosphorescence) originates from triplet excited states (^3^P_1_). In the same year, Majher et al. reported green emissions in Sb^3+^-doped In-based MHPs (Rb_3_InCl_6_) [85], as shown in Figure 5d. The distorted crystal structure of Rb_3_InCl_6_:Sb^3+^ results in a larger Stokes shift (1.29 eV) and lower energy emission (λmax = 522 nm) compared to the blue-emission Cs_2_NaInCl_6_:Sb^3+^. Recently, researchers reported some green emissions with highly efficient and stable Sb^3+^-doped Cd-based MHPs, which has attracted extensive attention. Xia et al. doped Sb^3+^ into Rb_4_CdCl_6_ and achieved a bright green emission peak at 525 nm with a PLQY of 70.2%, as is shown in Figure 5e. We report green-emission perovskites such as Sb^3+^-doped Rb_3_Cd_2_Cl_7_ [94] and Sb^3+^-doped Cs_3_Cd_2_Cl_7_ [49] (Figure 5f), both of which are produced by triplet STEs of Sb^3+^. The emission peak of Sb^3+^-doped Rb_3_Cd_2_Cl_7_ is 525 nm with a Stokes shift of 200 nm, and its PLQY is 57.47%. The emission peak of Sb^3+^-doped Cs_3_Cd_2_Cl_7_ is 517 nm, and its PLQY is 66%. These high-efficiency and stable green-emitting phosphors have great application potential in lighting, transportation, medicine, and other fields.

#### 3.4.3. Yellow Emission and Orange Emission

**Figure 6:**
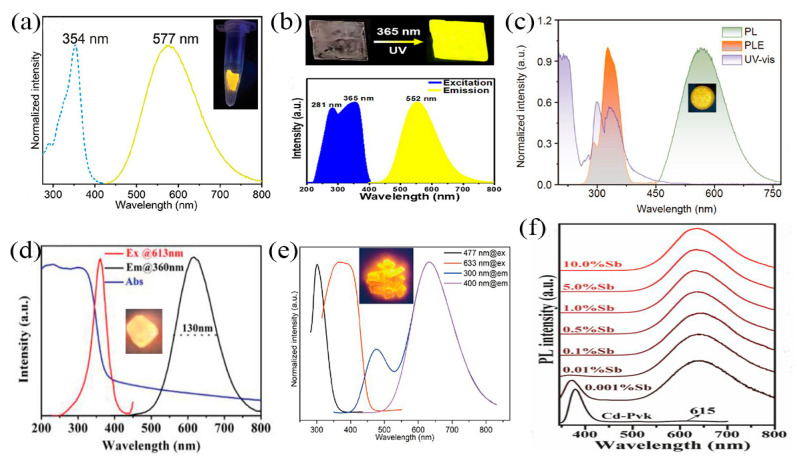
(**a**) PLE and PL spectra of [Emim]_8_[SbCl_6_]_2_[SbCl_5_]. Reprinted with permission from Ref. [103]. Copyright 2021, Royal Society of Chemistry. (**b**) PLE and PL spectra of β-[DHEP]SbCl_5_·2H_2_O. Reprinted with permission from Ref. [78]. Copyright 2022, American Chemical Society. (**c**) ABS, PL, and PLE spectra of C_6_H_18_N_2_)InCl_5_·H_2_O:Sb. Reprinted with permission from Ref. [104]. Copyright 2022, Springer. (**d**) ABS, PL, and PLE spectra of (C_6_N_2_H_16_)SbCl_5_. Reprinted with permission from Ref. [106]. Copyright 2020, Wiley. (**e**) PL and PLE spectra of (C_16_H_28_N)_2_SbCl_5_ SCs. Reprinted with permission from Ref. [71]. Copyright 2022, Springer. (**f**) PL spectra (λ_ex_ = 335 nm) of [NH_3_(CH_2_)_4_NH_3_]CdBr_4_-X%Sb. Reprinted with permission from Ref. [86]. Copyright 2021, American Chemical Society.

In our previous work, we found that Sb^3+^ exhibits yellow emission in low-dimensional structures [48,79]. Singlet and triplet emission bands can be observed in both (TMA)_2_SbCl_5_·DMF and Sb^3+^-doped RbCdCl_3_ under high-energy optical excitation. The high-energy emission band belongs to the ^1^P_1_–^1^S_0_ transition, and the low-energy emission band belongs to the ^3^P_n_–^1^S_0_ transition (where n = 0, 1, 2). In addition, there are numerous reports of similar phenomena. Zhang et al. fabricated novel 0D perovskite structures ([Emim]_8_[SbCl_6_]_2_[SbCl_5_]) by self-assembly of isolated [SbCl_5_]^2−^ pyramidal structures and [SbCl_6_]^3−^ octahedral structures with [Emim]^+^ cations [103]. [Emim]_8_[SbCl_6_]_2_[SbCl_5_]) has a broad yellow emission peak at 577 nm at an excitation wavelength of 354 nm, as shown in Figure 6a. Recently, Li et al. reported Sb-based MHPs (β-[DHEP]SbCl_5_·2H_2_O) with an emission peak of 552 nm (Figure 6b) and PLQY of 93.35% [78]. Its applications in information security, optical logic gates, and rewritable PL paper are proven. In addition, Shi et al. reported a highly emissive Sb^3+^-doped (C_6_H_18_N_2_)InCl_5_·H_2_O with an emission peak of 565 nm (Figure 6c) and a PLQY of 74.6% [104]. Interestingly, (C_6_H_18_N_2_)InCl_5_·H_2_O:Sb is a promising methanol detector. When it was exposed to methanol vapor, the emission peak shifted sharply from 565 nm to 663 nm, and after a few minutes in the air, the emission returned to 565 nm. These Sb-MHPs of yellow emission have shown great potential in the fields of solid-state lighting, anti-counterfeiting, and gas detection.

A large number of Sb-MHPs have been reported as a new orange emission source [105]. Xia et al. synthesized two Sb-based organic–inorganic hybrid MHPs ((C_6_N_2_H_16_)SbCl_5_ and (C_6_N_2_H_16_)SbCl_5_·H_2_O) by a simple method [106]. The two compounds exhibit similar broadband orange emissions under UV excitation. (C_6_N_2_H_16_)SbCl_5_ exhibits an orange emission peak at 613 nm under UV excitation at 360 nm with a PLQY of 25.3%, as shown in Figure 6d. The emission peak of (C_6_N_2_H_16_)SbCl_5_·H_2_O is red-shifted to 620 with a PLQY of 39.6%. They believe that the increase in PLQY is mainly due to the incorporation of water molecules, leading to more localized photoelectrons. Recently, we also reported orange emission of Sb-based MHPs ((C_16_H_28_N)_2_SbCl_5_ SCs) with a PLQY of 97%. Under UV excitation, (C_16_H_28_N)_2_SbCl_5_ exhibits a double emission band with an emission peak at 477 nm and 633 nm, which is generated by singlet and triplet STE emission bands, respectively, as shown in Figure 6e. In addition, orange emission was also reported in Sb^3+^-doped MHPs. A series of Sb^3+^-doped 2D [NH_3_(CH_2_)_4_NH_3_]CdBr_4_ by Wu et al. Efficient energy transfer between [NH_3_(CH_2_)_4_NH_3_]CdBr_4_ and Sb^3+^ dopant results in strong Sb^3+^ triplet emission and near-unity PLQY (Figure 6f). These orange emitters with excellent optical properties are of great significance for the study of MHPs.

#### 3.4.4. Red Emission and Near-Infrared Emission

**Figure 7:**
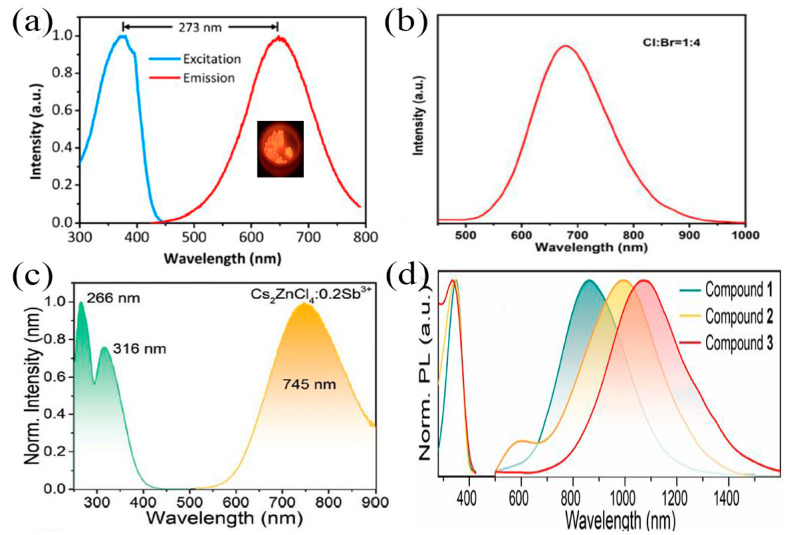
(**a**) Excitation and emission spectra of bulk (Ph_4_P)_2_SbCl_5_ crystals. Reprinted with permission from Ref. [107]. Copyright 2018, American Chemical Society. (**b**) Emission spectrum of Sb:Cs_2_InClBr_4_·H_2_O. Reprinted with permission from Ref. [108]. Copyright 2022, Elsevier. (**c**) Normalized PL and PLE spectra of 0.2Sb^3+^:Cs_2_ZnCl_4_. Reprinted with permission from Ref. [50]. Copyright 2021, Wiley. (**d**) Normalized RT PL and PLE spectra of 1 ((C_13_H_22_N)_2_Sb_2_Cl_8_), 2 ((C_10_H_16_N)_2_Sb_2_Cl_8_), and 3 ((C_16_H_36_P)Sb_2_Cl_8_) under 350, 345, and 335 nm excitation, respectively. Reprinted with permission from Ref. [89]. Copyright 2022, Wiley.

Red emission materials are widely used in display, transportation, lighting, medical treatment, and so on. Some studies have shown that Sb-MHPs have good application potential in the field of red emission materials [90]. For example, Ma et al. reported an efficient red-emitting (Ph_4_P)_2_SbCl_5_ bulk single crystal with an efficient broadband red emission band at 648 nm with a PLQY of about 87% [107], as shown in Figure 7a. Recently, we synthesized highly efficient and stable Sb:Cs_2_InClBr_4_·H_2_O crystals by anionic component engineering. The synthesized Sb:Cs_2_InClBr_4_·H_2_O crystal has a wide emission band for self-trapping excitons at 680 nm (Figure 7b) [108], and its photoluminescence quantum yield is as high as 93%. These Sb-MHPs of highly efficient red-emission have greatly facilitated the development of low-toxicity metal halide perovskites and their applications in optoelectronic devices.

The near-infrared light source has been widely used in medical treatment, biosensing, health monitoring, spectroscopy, and other fields because of its low thermal effect, non-destructive nature, and good penetration [109,110]. The current near-infrared fluorescent materials all face the advantages of low efficiency, short lifetime, poor stability or complexity, etc. [111]. MHP with excellent performance has great application potential in the field of luminescence. In 2021, Xia et al. reported near-infrared emission of Cs_2_ZnCl_4_:Sb^3+^ with an emission peak at 745 nm (Figure 7c) and a PLQY as high as 69.9% [50]. The broadband NIR emission comes from triplet STEs emission from doped [SbCl_4_]^−^ polyhedra (^3^P_1_ → ^1^S_0_). Cs_2_ZnCl_4_:Sb^3+^ has good air/thermal stability. When Cl^−^ is replaced by Br^−^, the luminescence peak is red-shifted from 745 nm to 823 nm. Recently, Xia et al. again reported three Sb-based MHPs with NIR emission, as shown in Figure 7d [89]. Compounds 1 ((C_13_H_22_N)_2_Sb_2_Cl_8_), 2 ((C_10_H_16_N)_2_Sb_2_Cl_8_), and 3 ((C_16_H_36_P)Sb_2_Cl_8_) exhibit wideband near-infrared emission peaks at 865, 990, and 1070 nm under excitation at 350, 345, and 335 nm, respectively. These NIR emissions are all from the triplet emission of Sb^3+^. These environmentally friendly and efficient near-infrared emission Sb-MHPs greatly expand the potential applications of metal-halide perovskites.

#### 3.4.5. White Emission

**Figure 8:**
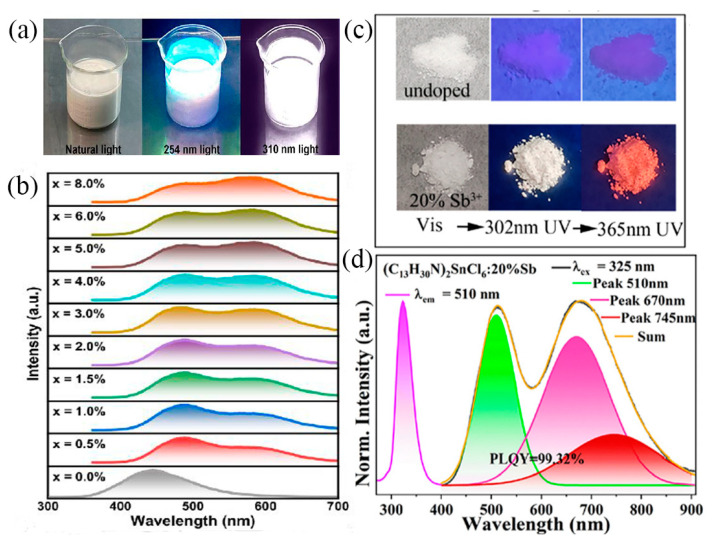
(**a**) Photographs of the Cs_2_ZrCl_6_:1.5%Sb under natural light and different UV lamps; (**b**) PL spectra of Cs_2_ZrCl_6_:xSb^3+^ (x = 0–8%). Reprinted with permission from Ref. [80]. Copyright 2022, American Chemical Society. (**c**) Photographs of undoped (C_13_H_30_N)_2_SnCl_6_ and (C_13_H_30_N)_2_SnCl_6_:20%Sb samples under visible light, 302 nm UV lamp, and 365 nm UV lamp, respectively. (**d**) Normalized PL spectrum of (C_13_H_30_N)_2_SnCl_6_:20%Sb excited at 325 nm and PLE spectrum excited at 510 nm. Reprinted with permission from Ref. [95]. Copyright 2023, Royal Society of Chemistry.

White emissive materials are considered to be an effective replacement for solid-state lighting, the next generation of lighting sources [112]. The general strategy is to display complementary light in the three primary colors (red, green, and blue). However, there are problems such as the non-uniformity of the luminescent properties of the compounds and different light attenuation and temperature-dependent properties [113]. Therefore, there is a need to find efficient single-composites of white emissive materials to generate white light. It was confirmed that Sb^3+^ with unique singlet and triplet excitons can exhibit blue and orange emission bands [114]. Therefore, singlet white light emission can be achieved by adjusting the singlet/triplet emission of Sb^3+^ ions. Shan et al. reported the dual emission band of Sb^3+^-doped Cs_2_ZrCl_6_ [80]. By changing the doping amount of Sb^3+^, the ratio of double emission bands can be adjusted to produce white light, whose PL spectrum is shown in Figure 8b. The best Cs_2_ZrCl_6_:1.5%Sb exhibited high-quality white light (Figure 8a) with a CRI of 96 and a PLQY of 52.48%. In addition, Lin et al. introduced %1.5Sb^3+^ into PA_6_InCl_9_ to obtain a white phosphor with nearly 100% PLQY and a color rendering index (CRI) close to 90 [115]. Recently, we reported a white luminescent material based on (C_13_H_30_N)_2_SnCl_6_:20%Sb [95], which generates strong white emission under 325 nm excitation and exhibits nearly 100% PLQY. These studies are bound to provide new insights into the preparation of single-component white phosphors for next-generation lighting technologies.

### 3.5. The Tunable Emission of Sb-Activated MHPs

**Figure 9:**
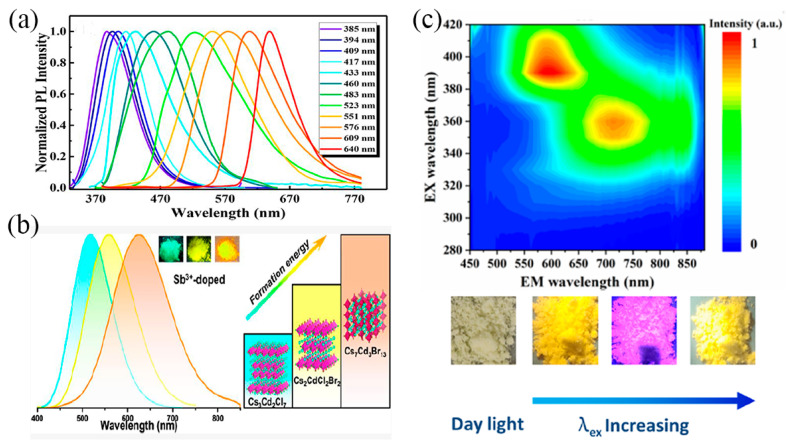
(**a**) PL spectra of Cs_3_Sb_2_X_9_ QDs (X = Cl_x_Br_y_I_1−x−y_, 0 ≤ x, y ≤ 1). Reprinted with permission from Ref. [93]. Copyright 2020, American Chemical Society. (**b**) PL spectra of Sb^3+^-doped Cs_3_Cd_2_Cl_7_, Cs_2_Cd_2_Cl_2_Br_2_, and Cs_7_Cd_3_Cl_13_. Reprinted with permission from Ref. [49]. Copyright 2022, American Chemical Society. (**c**) Excitation-dependent PL spectra of 5%Sb^3+^:(NH_4_)_2_SnCl_6_. Reprinted with permission from Ref. [116]. Copyright 2023, American Chemical Society.

The ability of halogen-substituted anion exchange reactions to modulate emission bands has been widely reported in CsPbX_3_ (X = Cl, Br, and I) [23,60]. Such halogen ion substitution reaction-tuning emission bands have also been widely reported in Sb-MHPs. Song et al. obtained Cs_3_Sb_2_X_9_ QDS by anion exchange reaction; the luminescence wavelength can be adjusted from 370 nm to 560 nm [31]. Zhao et al. reported all-inorganic double perovskite Cs_2_AgSbX_6_ quantum dots [67]. Changing the precursor’s halogen composition, the PL emission wavelength was tuned from 409 nm to 557 nm. Subsequently, Shan et al. synthesized Cs_3_Sb_2_X_9_ quantum dots by the LARP method [93]. After in-depth research, they found that the halogen-rich surface of NCs has a quantum well structure, which is conducive to efficient radiative transitions. In addition, the emission spectrum of the product was adjusted by changing the composition of the halide, and the tunable wavelength range was 385–640 nm, as shown in Figure 9a. Tunability is significantly better than other lead-free perovskite quantum dots. Compositional variability enables further tunable optical properties of Sb-MHPs. Our previous work reported that the tunable emission of Sb-MHPs was achieved by modulating the composition. For example, Huang et al. achieved tunable green-yellow emission by controlling the structural transformation of Sb-doped indium halides A_3_InCl_6_ and A_2_InCl_5_∙H_2_O (A = Cs, Rb) [47]. Recently, Chang explored a phase-selective solution synthesis route to obtain a series of different Cd-based perovskite derivatives doped with Sb^3+^ [49]. Combined with the halogen substitution method, a series of Cd-based perovskite derivatives with different crystal structures and emission spectra (517~625 nm) were obtained, which can achieve tunable emission from cyan to orange, as is shown in Figure 9b. In addition, by introducing Sb^3+^ ions into (NH_4_) _2_SnC_l6_ through a doping strategy, we achieved for the first time a change in excitation light from 360 nm to 390 nm at room temperature, making the emission dynamically tunable from yellow to near infrared (NIR) [116]. A hybrid STE with Sb and host is proposed for the first time to emit tuned luminescence.

### 3.6. Summarization of the Separated

#### 3.6.1. Dimensionality

As mentioned above, Sb^3+^ activated luminescent metal halide bulk materials typically exhibit broadband STE emission. In metal halide materials, the formation of localized self-trapped excited states is closely related to their structural dimensions, and reducing the dimensions will make it easier for excitons to self-trap. The quantum confinement effect in the 0D structure is strong, and STE produces efficient luminescence with a particularly high PLQY. For example, the PLQY of 0D-(MePPh_3_)_2_SbCl_5_ is 99.4% [117]. There are few reports on 1D-chain Sb-based metal halides. In 2021, Zhang et al. reported two 1D chain-shaped Sb-based metal halides, (2cepyH)SbCl_4_ and (2cepyH)SbBr_4_ [118]. No emission behavior was observed at room temperature for (2cepyH) SbBr_4_. (2cepyH) SbCl_4_ exhibits yellow emission at 570 nm at room temperature, with a FWHM of 115 nm and a lifetime of 6.85 μs. This is the first time broadband emissions have been observed in one-dimensional antimony-based hybrid materials. This emission behavior is basically consistent with the triplet STE emission behavior of Sb-based metal halides in 0D. But the PLQY of (2cepyH) SbCl_4_ is very low, only 4.5%, which may be due to severe concentration quenching in one-dimensional chain structures. Currently, the reported 2D antimony-based metal halides are all inorganic, such as Rb_7_Sb_3_Cl_16_, Rb_3_Sb_2_I_9_, etc. [90,91]. These bulk materials exhibit broadband STE emission, with emission behavior consistent with 0D-Sb-based halides, but PLQY is relatively low (Rb_7_Sb_3_Cl_16_ 26%).

#### 3.6.2. A-Position Cation

**Figure 10:**
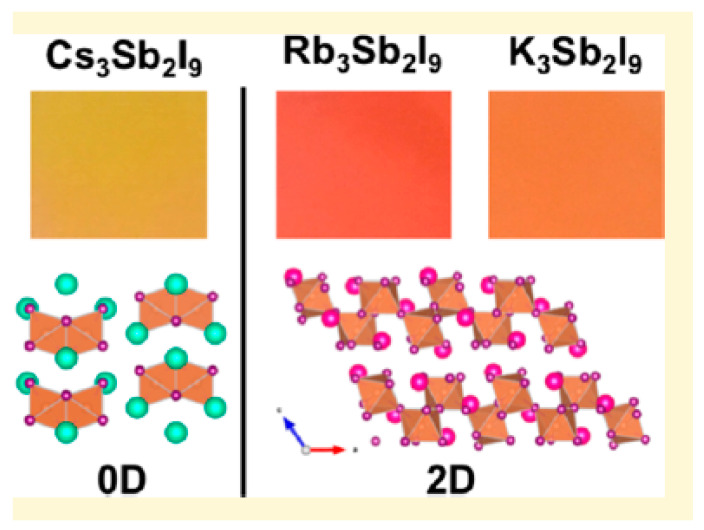
Crystal structures of A_3_Sb_2_I_9_ (A = Cs, Rb, K). Reprinted with permission from Ref. [70]. Copyright 2018, American Chemical Society.

Previously, Buonassisi et al. reported on the effect of A-site cations on all-inorganic Sb-based metal halides and found that A-site cations dominate the structure and photoelectric properties of these compounds [70]. Cs_3_Sb_2_I_9_ has a 0D structure and an indirect bandgap (Figure 10). Rb_3_Sb_2_I_9_ has a 2D structure and a direct bandgap. K_3_Sb_2_I_9_ has a 2D structure and an indirect bandgap. The ion radius of Cs^+^ is greater than Rb^+^ and K^+^, and Cs_3_Sb_2_I_9_ tends to form a 0D structure. However, the PLQY of these compounds is very low, which may be due to the small size of A-site ions. Due to the large size of organic cations, most of the reported organic-inorganic hybrid Sb-based metal halides have a 0D structure [37]. Compared to all-inorganic Sb-based metal halides, the PLQY of organic-inorganic hybrid Sb-based metal halides is much higher, some even close to 100% [117].

#### 3.6.3. Coordination

The coordination of Sb^3+^ ions in Sb^3+^-based hybrid metal halides can affect emission, and different coordination structures can be found in different compounds. The 4-coordinated seesaw (SbX_4_), 5-coordinated pyramid (SbX_5_), and 6-coordinated octahedron (SbX_6_) are relatively common. Mao et al. conducted extensive experiments on 4-coordinated and 5-coordinated Sb-based metal halides [119]. They found that 4-coordinated Sb-based metal halides did not exhibit emission behavior at room temperature, while 5-coordinated Sb-based metal halides had a high PLQY (>90%). Kundu et al. successfully synthesized the SbX_6_ compound C_12_H_52_Cl_18_N_8_O_4_Sb_3_ and the SbX_5_ compound C_12_H_50_Cl_14_N_8_O_3_Sb_2_ by controlling the ratio [102]. The former exhibits green emission (517 nm), while the latter exhibits yellow emission (590 nm). V. Kovalenko et al. also reported a similar situation, where [C@Ba]_4_[SbCl_6_]_2_[Sb_2_Cl_8_] with SbX_6_ exhibited green emission (550 nm), while [C@Cs]_2_CsSbBr_6_ with SbX_5_ exhibited yellow emission (600 nm) [120]. SbX_5_ typically exhibits low energy emissions, while SbX_6_ exhibits relatively high energy and green emissions.

### 3.7. Summary of Luminescence Performance of Sb-Activated MHPs

In summary, the optical properties of Sb^3+^ show great differences among Sb-MHPs with different structures. Table 1 summarizes a large number of existing Sb-MHPs and their optical parameters. As mentioned above, the unique singlet and triplet exciton emission mechanisms of Sb^3+^ can realize tunable emission and single-color emission in a wide range of visible to near-infrared wavelengths. Sb-MHPs with excellent optical properties and environmental friendliness will have the opportunity to replace toxic lead halide perovskite materials and have great application potential in solid-state lighting, LED, anti-counterfeiting, temperature sensing, photodetection, and other fields. However, what factors affect the triplet and singlet states of Sb^3+^ and the relationship between them is still unclear.

## 4. Applications

### 4.1. Light Emitting Diode (LED)

#### 4.1.1. White LED

**Figure 11:**
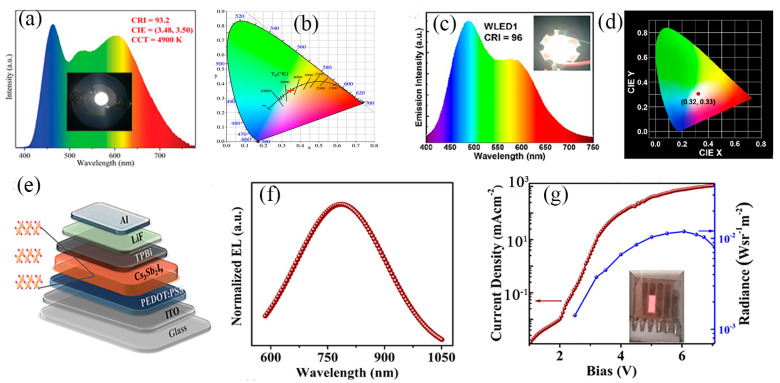
(**a**) Emission spectrum, and (**b**) CIE coordinates of (TPA)_2_SbCl_5_-based WLED. Reprinted with permission from Ref. [75]. Copyright 2021, Royal Society of Chemistry. (**c**) Emission spectrum, and (**d**) CIE color coordinate of Cs_2_ZrCl_6_:1.5%Sb-based WLED. Reprinted with permission from Ref. [80]. Copyright 2022, American Chemical Society. (**e**) Device architecture, (**f**) Normalized EL spectrum, and (**g**) Current density versus voltage (J–V) and radiance versus voltage characteristics of the Cs_3_Sb_2_I_9_ film. Reprinted with permission from Ref. [69]. Copyright 2019, American Chemical Society.

At present, blue wafers (InGaN) coated with yellow phosphor (YAG:Ce^3+^) are widely used to produce commercial WLEDs [121]. Here, WLEDs with a high color rendering index using different Sb-MHPs as luminescent layers are reviewed. In our previous report, we fabricated WLEDs by mixing orange-emission Cs_2_InCl_5_·H_2_O:Sb^3+^ with commercial blue (Ba_2_SiO_4_:Eu^2+^) and green (BaMgAl_10_O_17_:Eu^2+^) phosphors, as shown in Figure 11a [75]. The CIE coordinates of the prepared WLED are (3.48, 3.50) (Figure 11b), the color rendering index CRI is 93.2, and the CCT is 4900 K. The CIE coordinates located in the white light region and the ultra-high CRI demonstrate that (TPA)_2_SbCl_5_ is a promising down-conversion phosphor for WLED. However, this strategy of mixing several phosphors to obtain white light emission cannot achieve both color rendering and color stability. Recently reported as a one-component phosphor (Cs_2_ZrCl_6_:1.5%Sb) with very superior performance with broadband white emission [80], WLED was prepared by using a 310 nm excitation chip, as shown in Figure 11c,d. WLED exhibits bright white emission with CIE coordinates of (0.32, 0.33) and a record CRI of (96). In addition, T_50_ is confirmed to be ~2003 h, which is higher than all previously reported unleaded perovskite WLEDs. However, finding reasonable strategies to improve the stability of Sb-MHP phosphors is of great significance for the commercialization of Sb-MPH-based WLED devices.

#### 4.1.2. Electroluminescent LEDs

For Sb-MHPs-based electroluminescent LEDs (Sb-PLEDs), the highly emissive perovskite layer is only one component required and typically also has an anode, a hole transport layer (HTL), an emission layer (EML), an electron transport layer (ETL), and a cathode. The device structure is similar to solution-processed organic LEDs (OLEDs) and conventional quantum dot-based LEDs (QLEDs) [2]. When a voltage is applied to the device, holes and electrons are injected from the anode and cathode and enter the EML through the HTL and ETL, where they form excitons, followed by efficient radiative recombination to emit photons [122]. Chu et al. reported a red Sb-LED based on Cs_3_Sb_2_I_9_ [69], the structure of which is shown in Figure 11e. Under the current condition, the electroluminescence spectrum of LED becomes a broadband red spectrum (Figure 11f). When the voltage is 6v, the radiance reaches its highest, which is 0.012 W·sr^−1^·m^−2^ (Figure 11g). Recent advances in Sb-PLEDs show their bright future in displays, lighting, and optical communications. However, as a young technology, there is still a gap in the operational stability and efficiency of Sb-PLEDs, which is a challenge for their practical application.

### 4.2. Temperature Sensor

**Figure 12:**
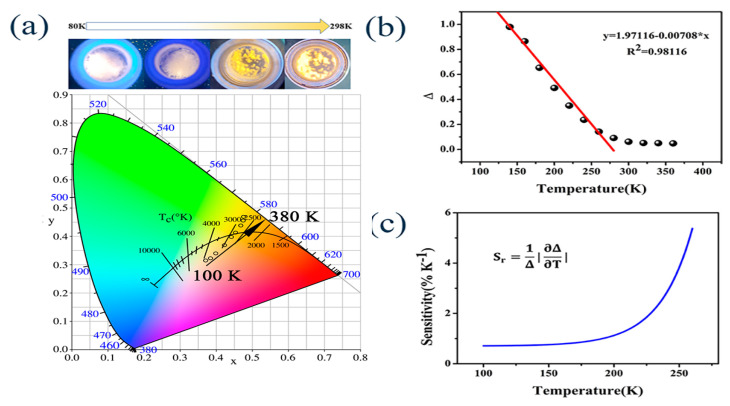
(**a**) CIE color coordinates in the interval 100 to 380 k; (**b**) temperature dependence of the emission intensity ratio of 464 to 600 nm; and (**c**) relative thermal sensitivity in the 100–260 K range of 40%Sb:RbCdCl_3_. Reprinted with permission from Ref. [79]. Copyright 2022, American Chemical Society.

Previously, a large number of reports have shown that the emission color of Sb-MHPs changes significantly with temperature [48,71]. This is mainly because the relative intensities of singlet STEs and triplet STEs of Sb^3+^ change with temperature, and these emission color changes can be used for temperature sensing. In the Sb-based metal halide system, the emission of singlet and triplet STE is closely related to the strength of electron-phonon coupling. Interestingly, the sensitivity of these two STE emission bands to the strength of electron–phonon coupling is usually different. As the temperature changes, the strength of electron phonon coupling also changes, and the changes in STE emission behavior of singlet and triplet states are usually inconsistent [79]. Usually, singlet STEs exhibit strong emissions at low temperatures. As the temperature increases, the PL peak shifts blue and the intensity decreases due to the intensified acoustic phonon exciton interaction and thermal expansion [92]. Until now, luminescence thermometers have mostly been based on the relationship between temperature and the luminescence intensity of a single luminescence transition [123], while Sb^3+^ can determine the temperature by the intensity ratio of the two luminescence transitions. In 2019, Fei et al. reported a strong luminescent material based on Sb^3+^-based coordination polymer [124], and they verified the linear relationship between the correlated color temperature and the absolute temperature in the range of 157~457 K, thus proposing the first photoemission energy-based Sb^3+^ based solid-state luminescent thermometer. Recently, we reported a temperature-sensitive Sb:RbCdCl_3_ material [79]. Figure 12a shows the pictures and color coordinates of 40% Sb:RbCdCl_3_ at different temperatures under UV excitation. It can be seen that the emission color changes significantly with temperature, which shows its application potential in temperature detection. The emission intensity ratio from 464 to 600 nm decreased linearly with temperature in the range of 100–260 K (Figure 12b). Figure 12c shows the sensitivity versus stability curve, which shows a maximum of 6% at 260 K, illustrating the possibility of using it as a luminescence thermometer in this temperature range.

### 4.3. Optical Anti-Counterfeiting Technology

**Figure 13:**
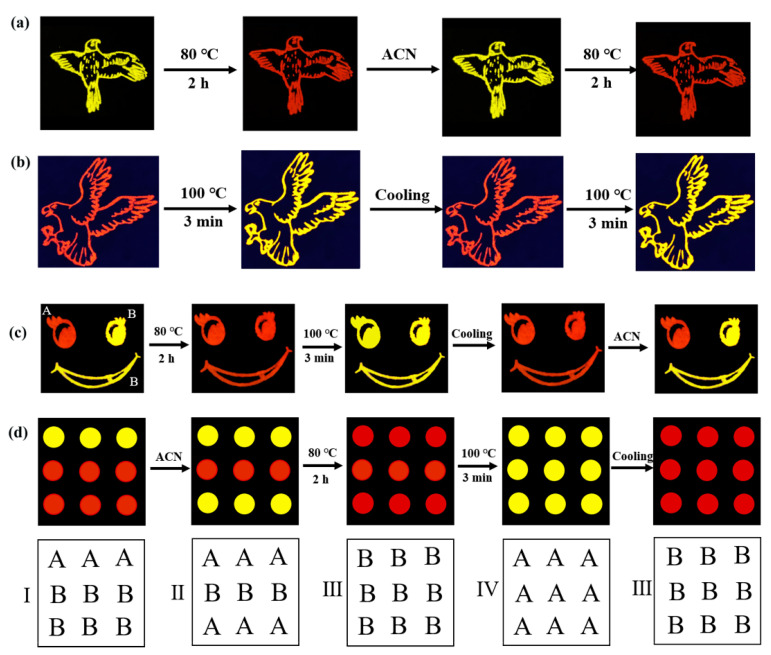
Anti-counterfeiting applications: image transitions of luminescence security patterns under 365 nm UV light based on [Ph_3_S]_2_SbCl_5_·2C_2_H_3_N (yellow color) (**a**), [Ph_3_S]_2_SbCl_5_ (Red color) (**b**), and combined [Ph_3_S]_2_SbCl_5_@epoxy resin and [Ph_3_S]_2_SbCl_5_·2C_2_H_3_N (**c**); (**d**) Illustration of the information encryption processes (I–IV represents the four types of information above, with A representing yellow color and B representing red color.). Reprinted with permission from Ref. [125]. Copyright 2022, Elsevier.

In addition, in the field of luminescence, Sb-MHPs also have great development potential in the field of optical anti-counterfeiting technology. Lei et al. fabricated a three-mode reversible PL switch anti-counterfeiting technique based on three Sb-based MHPs [78], including non-emitting α-[DHEP]SbCl_5_ (1), yellow-emitting β-[DHEP]SbCl_5_·2H_2_O (2), and red-emitting β-[DHEP]SbCl_5_ (3) three compounds, where the reversible transition between 1 and 2 is triggered by acetone or methanol, enabling reversible PL on-off switching (yellow). Recently, we have also done some original work in this direction. By inserting the organic ligand Ph_3_SCl (triphenylsulfonium chloride) into the SbCl_3_ lattice, we have obtained two different zero-dimensional (0D) organic antimony chlorides, [Ph3_S_]_2_SbCl_5_·2C_2_H_3_N and [Ph_3_S]_2_SbCl_5_ [125]. The dynamic transformation process between the compounds [Ph3_S_]_2_SbCl_5_·2C_2_H_3_N and [Ph_3_S]_2_SbCl_5_ can be completed by stripping/inserting acetonitrile (C_2_H_3_N) under different external stimuli. Continuous heating of [Ph_3_S]_2_SbCl_5_ causes the emission color to change again from red to yellow. Based on this process, we build a variety of anti-counterfeiting and information encryption models, as shown in Figure 13. In addition, we also report a kind of Sb^3+^ doped hexagonal CsCdCl_3_ anti-counterfeiting material with ultra-long afterglow emission [126]. Color-reconcilable and time-dependent ultra-long afterglow emission can be achieved by adjusting the doping concentration of Sb^3+^. In view of this unusual afterglow emission property, we built an optical anti-counterfeiting and information encryption system.

### 4.4. Scintillators

**Figure 14:**
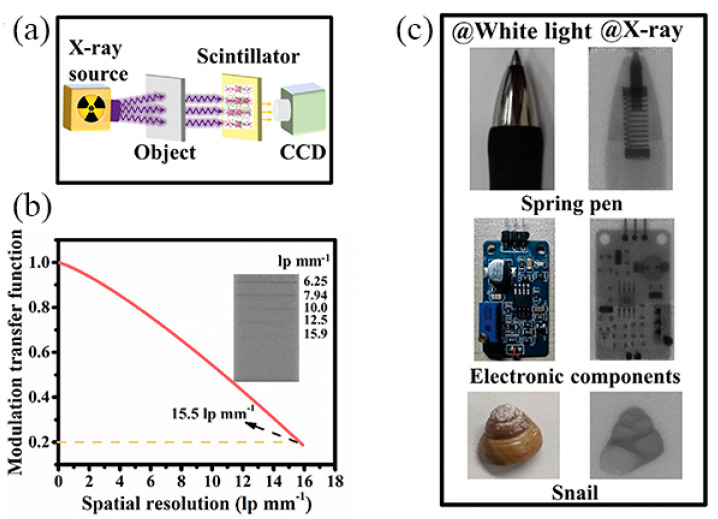
(**a**) Schematic of the experimental apparatus for X-ray imaging, (**b**) The spatial resolution of [Na(DMSO)_2_]_3_SbBr_3_Cl_3_@PMMA films is 15.5 lp mm^−1^ @ MTF = 0.2, (**c**) The images of objects under both white and X-ray light. Reprinted with permission from Ref. [128]. Copyright 2022, Wiley.

The scintillator is a material that luminesces after absorbing high-energy particles or rays. Sb-MHPs also make them an efficient X-ray scintillator due to the advantages of a simple synthesis process, low cost, and large Stokes shift. In 2021, Manna et al. reported scintillators based on 0.7% Sb-doped Cs_2_NaInCl_6_ and 0.9% Sb-doped Cs_2_KInCl_6_ NCs. The RL spectra are dominated by peaks at ~2.7 and ~2.4 eV, respectively, which are very similar to their respective PL emissions, demonstrating that the same excited states as UV excitations are generated under X-ray irradiation, indicating that these two materials are promising candidates for scintillation applications [127]. Recently, Zang et al. reported an X-ray scintillator based on [Na(DMSO)_2_]_3_SbBr_3_Cl_3_ [128]. To further verify the application of X-ray scintillators, a schematic diagram of the experimental setup as shown in Figure 14a was established. The spatial resolution of the [Na(DMSO)_2_]_3_SbBr_3_Cl_3_@PMMA film is 15.5 lp mm^−1^ @ MTF = 0.2 (Figure 14b). Figure 14c shows an image of a spring pin, electronics, and snail as targets under white light and X-ray illumination, which nicely shows the fine details inside the sample. This work demonstrates the potential of Sb-MHPs for X-ray imaging.

## 5. Summary and Perspectives

In conclusion, Sb-MHPs can overcome the inherent toxicity of lead halide perovskites, and great progress has been made in various aspects. However, there are still some problems, and there are still many challenges in many aspects. In this paper, the structure and luminescence mechanism of Sb^3+^ are summarized, the synthesis methods and basic luminescence properties of various types of Sb-MHPs are reviewed, and the applications in WLED, electroluminescence LED, temperature sensing, optical anti-counterfeiting, and X-ray scintillation are discussed. We believe that further theoretical and experimental breakthroughs are needed to develop Sb-MHPs with high performance and stability. We present the following challenges:

(1) At present, there is a lack of theoretical guidance in the development of novel Sb-MHPs, including whether we can use theoretical calculations to predict how various materials self-assemble to form novel Sb-MHPs. In addition, the influencing factors of the singlet and triplet double emission bands of Sb^3+^ are still unclear, and more theoretical research is needed to understand this optical property.
(2) As ionic compounds, Sb-MHPs are physically and chemically unstable in environments such as heat, humidity, and light. Especially in lighting applications, the excitation threshold increases significantly with increasing temperature, which requires the rational design of calcium Sb-MHPs with temperature insensitivity. Further research is needed to further improve its stability and realize its industrial production and application as soon as possible.
(3) It is necessary to develop a variety of synthetic routes to develop efficient, stable, and multifunctional Sb-MHPs and to introduce more application fields, such as fluorescence sensors, lasers, and other fields.

Although Sb-MHPs still have a long way to go on the road to commercialization, this material has demonstrated strong potential in optoelectronic applications and is very promising as a next-generation light source. We hope that this review can deepen the understanding of Sb-MHPs in the field.

## Figures and Tables

**Table 1:** Comparison of Optical properties of Sb-based inorganic metal halide perovskites.

Chemical Formula	PLE/PL Peak (nm)	Color	FWHM (nm)	PLQY	Lifetime (ns)
Cs_3_Sb_2_Cl_9_ QDs [31]	/370	violet	52	11%	2.96
Cs_2_AgSbCl_6_ QDs [67]	325/409	blue	70	31.33%	10.09
Cs_3_Sb_2_Br_9_ QDs [31]	375/410	blue	41	46%	4.29
Cs_2_NaInCl_6_:5%Sb [99]	330/445	blue	~80	79%	1029
Cs_2_AgSbBr_6_ NCs [67]	440/465	blue	82	0.7%	
14%Sb^3+^:Cs_2_KInCl_6_ [101]	290/495	cyan	~95	99.2%	2990
Cs_2_CdCl_4_:5%Sb^3+^ NPs [81]	280/510	cyan	103	20%	1625.9
Sb^3+^:(Cs_1−x_Rb_x_)_3_InCl_6_ [47]	320/513	cyan	~105	91.8%	3090
C_12_H_52_Cl_18_N_8_O_4_Sb_3_ [102]	360/517	green	110	45%	4800
20%Sb^3+^:Rb_3_Cd_2_Cl_7_ [94]	325/525	green	115	57.47%	19,400
0.4%Sb^3+^:Rb_4_CdCl_6_ [77]	340/525	green	116	70%	1845
Cs_3_Sb_2_Br_0.5_I_0.5_ QDs [67]	/531	green		90%	
Sb:Cs_2_CdCl_2_Br_2_ [49]	355/559	yellow	123	35.23%	2140
β-[DHEP]SbCl_5_·2H_2_O [78]	365/552	yellow	122	93.35	10,550
Rb_7_Sb_3_Cl_16_ NCs [65]	325/556	yellow	101	≤1%	
[Emim]_8_[SbCl_6_]_2_[SbCl_5_] [103]	354/577	yellow	146	11.2%	2250
C_24_H_88_Cl_25_N_16_O_4_Sb_3_ [102]	360/590	yellow	140	43%	5200
Rb_7_Sb_3_Br_16_ SCs [105]	350/600	orange	≈95		
(TPA)_2_SbCl_5_ [75]	375/610	orange	130	95.3%	5300
Sb:Cs_7_Cd_3_Br_13_ [49]	375/625	orange	147	57.42%	2170
Cs3Sb2I9 SCs [90]	473/632	red	94		
Rb3Sb2I9 SCs [90]	473/646	red	117		
C_12_H_50_Cl_14_N_8_O_3_Sb_2_ [102]	340/650	red	160	6%	5600
Sb:Cs_2_InClBr_4_·H_2_O [108]	380/680	red	180	93%	3310
Sb^3+^:Cs_2_ZnCl_4_ [50]	316/745	near-infrared	175	69.9%	12,800
(C_13_H_22_N)_2_Sb_2_Cl_8_ [89]	350/865	near-infrared	~230	5%	1870
(C_10_H_16_N)_2_Sb_2_Cl_8_ [89]	345/990	near-infrared	~260	3%	1580
(C_16_H_36_P)SbCl_4_ [89]	335/1070	near-infrared	~280	1%	1340
Cs_2_ZrCl_6_:1.5%Sb [80]	310/480,580	white		52.48%	5.62/61.64
PA_6_InCl_9_-Sb [115]	282/468,580	white		90.6%	
(C_13_H_30_N)_2_SnCl_6_:20%Sb [95]	325/510,670	white		99.32%	23.48/7760

## References

[1] Lee J.W., Tan S., Seok S.I., Yang Y., Park N.G. (2022). Rethinking the A cation in halide perovskites. Science.

[2] Liu X.K., Xu W., Bai S., Jin Y., Wang J., Friend R.H., Gao F. (2021). Metal halide perovskites for light-emitting diodes. Nat. Mater..

[3] Choi Y.C., Jung K.W. (2020). Recent Progress in Fabrication of Antimony/Bismuth Chalcohalides for Lead-Free Solar Cell Applications. Nanomaterials.

[4] Su B., Zhou G., Huang J., Song E., Nag A., Xia Z. (2020). Mn^2+^-Doped Metal Halide Perovskites: Structure, Photoluminescence, and Application. Laser Photonics Rev..

[5] Wells H.L. (1893). On the caesium- and the potassium-lead halides. Am. J. Sci..

[6] Kojima A., Teshima K., Shirai Y., Miyasaka T. (2009). Organometal halide perovskites as visible-light sensitizers for photovoltaic cells. J. Am. Chem. Soc..

[7] Huang T., Peng H., Wei Q., Peng C., Tian Y., Yao S., Han X., Zou B. (2022). Magnetic polaronic and bipolaronic excitons in Mn(II) doped (TDMP)PbBr_4_ and their high emission. Nano Energy.

[8] Shamsi J., Urban A.S., Imran M., De Trizio L., Manna L. (2019). Metal Halide Perovskite Nanocrystals: Synthesis, Post-Synthesis Modifications, and Their Optical Properties. Chem. Rev..

[9] Wu Y., Fan W., Gao Z., Tang Z., Lei L., Sun X., Li Y., Cai H.-L., Wu X. (2020). New photoluminescence hybrid perovskites with ultrahigh photoluminescence quantum yield and ultrahigh thermostability temperature up to 600 K. Nano Energy.

[10] Wang C., Han D., Wang J., Yang Y., Liu X., Huang S., Zhang X., Chang S., Wu K., Zhong H. (2020). Dimension control of in situ fabricated CsPbClBr_2_ nanocrystal films toward efficient blue light-emitting diodes. Nat. Commun..

[11] Tan Z.K., Moghaddam R.S., Lai M.L., Docampo P., Higler R., Deschler F., Price M., Sadhanala A., Pazos L.M., Credgington D. (2014). Bright light-emitting diodes based on organometal halide perovskite. Nat. Nanotechnol..

[12] Liu M., Wan Q., Wang H., Carulli F., Sun X., Zheng W., Kong L., Zhang Q., Zhang C., Zhang Q. (2021). Suppression of temperature quenching in perovskite nanocrystals for efficient and thermally stable light-emitting diodes. Nat. Photonics.

[13] Zhou G., Jiang X., Molokeev M., Lin Z., Zhao J., Wang J., Xia Z. (2019). Optically Modulated Ultra-Broad-Band Warm White Emission in Mn^2+^-Doped (C_6_H_18_N_2_O_2_)PbBr_4_ Hybrid Metal Halide Phosphor. Chem. Mater..

[14] Li M., Zhou J., Zhou G., Molokeev M.S., Zhao J., Morad V., Kovalenko M.V., Xia Z. (2019). Hybrid Metal Halides with Multiple Photoluminescence Centers. Angew. Chem. Int. Ed..

[15] Huang Y., Qiao L., Jiang Y., He T., Long R., Yang F., Wang L., Lei X., Yuan M., Chen J. (2019). A-site Cation Engineering for Highly Efficient MAPbI_3_ Single-Crystal X-ray Detector. Angew. Chem. Int. Ed..

[16] He T., Li S., Jiang Y., Qin C., Cui M., Qiao L., Xu H., Yang J., Long R., Wang H. (2020). Reduced-dimensional perovskite photovoltaics with homogeneous energy landscape. Nat. Commun..

[17] Wu X., Ji H., Yan X., Zhong H. (2022). Industry outlook of perovskite quantum dots for display applications. Nat. Nanotechnol..

[18] Xing G., Mathews N., Lim S.S., Yantara N., Liu X., Sabba D., Gratzel M., Mhaisalkar S., Sum T.C. (2014). Low-temperature solution-processed wavelength-tunable perovskites for lasing. Nat. Mater..

[19] Tian W., Zhou H., Li L. (2017). Hybrid Organic-Inorganic Perovskite Photodetectors. Small.

[20] Parobek D., Roman B.J., Dong Y., Jin H., Lee E., Sheldon M., Son D.H. (2016). Exciton-to-Dopant Energy Transfer in Mn-Doped Cesium Lead Halide Perovskite Nanocrystals. Nano Lett..

[21] Huang G., Wang C., Xu S., Zong S., Lu J., Wang Z., Lu C., Cui Y. (2017). Postsynthetic Doping of MnCl_2_ Molecules into Preformed CsPbBr_3_ Perovskite Nanocrystals via a Halide Exchange-Driven Cation Exchange. Adv. Mater..

[22] Zhang M., Zheng Z., Fu Q., Chen Z., He J., Zhang S., Yan L., Hu Y., Luo W. (2017). Growth and characterization of all-inorganic lead halide perovskite semiconductor CsPbBr_3_ single crystals. CrystEngComm.

[23] Peng L., Dutta S.K., Mondal D., Hudait B., Shyamal S., Xie R., Mahadevan P., Pradhan N. (2019). Arm Growth and Facet Modulation in Perovskite Nanocrystals. J. Am. Chem. Soc..

[24] Deng C., Zhou G., Chen D., Zhao J., Wang Y., Liu Q. (2020). Broadband Photoluminescence in 2D Organic-Inorganic Hybrid Perovskites: (C_7_H_18_N_2_)PbBr_4_ and (C_9_H_22_N_2_)PbBr_4_. J. Phys. Chem. Lett..

[25] Navas J., Sanchez-Coronilla A., Gallardo J.J., Hernandez N.C., Pinero J.C., Alcantara R., Fernandez-Lorenzo C., De los Santos D.M., Aguilar T., Martin-Calleja J. (2015). New insights into organic-inorganic hybrid perovskite CH_3_NH_3_PbI_3_ nanoparticles. An experimental and theoretical study of doping in Pb^2+^ sites with Sn^2+^, Sr^2+^, Cd^2+^ and Ca^2+^. Nanoscale.

[26] Liu X., Xu X., Li B., Yang L., Li Q., Jiang H., Xu D. (2020). Tunable Dual-Emission in Monodispersed Sb^3+^/Mn^2+^ Codoped Cs_2_NaInCl_6_ Perovskite Nanocrystals through an Energy Transfer Process. Small.

[27] Roccanova R., Yangui A., Nhalil H., Shi H., Du M.-H., Saparov B. (2019). Near-Unity Photoluminescence Quantum Yield in Blue-Emitting Cs_3_Cu_2_Br_5−x_I_x_ (0 ≤ x ≤ 5). ACS Appl. Electron. Mater..

[28] Huang S., Li Z., Kong L., Zhu N., Shan A., Li L. (2016). Enhancing the Stability of CH_3_NH_3_PbBr_3_ Quantum Dots by Embedding in Silica Spheres Derived from Tetramethyl Orthosilicate in “Waterless” Toluene. J. Am. Chem. Soc..

[29] Zhao X., Tan Z.-K. (2019). Large-area near-infrared perovskite light-emitting diodes. Nat. Photonics.

[30] Luo J., Yang L., Tan Z., Xie W., Sun Q., Li J., Du P., Xiao Q., Wang L., Zhao X. (2021). Efficient Blue Light Emitting Diodes Based On Europium Halide Perovskites. Adv. Mater..

[31] Zhang J., Yang Y., Deng H., Farooq U., Yang X., Khan J., Tang J., Song H. (2017). High Quantum Yield Blue Emission from Lead-Free Inorganic Antimony Halide Perovskite Colloidal Quantum Dots. ACS Nano.

[32] Xu L., Gao J.X., Chen X.G., Hua X.N., Liao W.Q. (2018). A temperature-triggered triplex bistable switch in a hybrid multifunctional material: [(CH_2_)_4_N(CH_2_)_4_]_2_[MnBr_4_]. Dalton Trans..

[33] Su B., Song G., Molokeev M.S., Lin Z., Xia Z. (2020). Synthesis, Crystal Structure and Green Luminescence in Zero-Dimensional Tin Halide (C_8_H_14_N_2_)_2_SnBr_6_. Inorg. Chem..

[34] Zhao X.G., Yang J.H., Fu Y., Yang D., Xu Q., Yu L., Wei S.H., Zhang L. (2017). Design of Lead-Free Inorganic Halide Perovskites for Solar Cells via Cation-Transmutation. J. Am. Chem. Soc..

[35] Yin W.J., Shi T., Yan Y. (2014). Unique properties of halide perovskites as possible origins of the superior solar cell performance. Adv. Mater..

[36] Zhou G., Su B., Huang J., Zhang Q., Xia Z. (2020). Broad-band emission in metal halide perovskites: Mechanism, materials, and applications. Mater. Sci. Eng. R Rep..

[37] Jing Y., Liu Y., Li M., Xia Z. (2021). Photoluminescence of Singlet/Triplet Self-Trapped Excitons in Sb^3+^-Based Metal Halides. Adv. Opt. Mater..

[38] Pal J., Manna S., Mondal A., Das S., Adarsh K.V., Nag A. (2017). Colloidal Synthesis and Photophysics of M_3_Sb_2_I_9_ (M = Cs and Rb) Nanocrystals: Lead-Free Perovskites. Angew. Chem. Int. Ed..

[39] Mahmood Q., Hassan M., Yousaf N., AlObaid A.A., Al-Muhimeed T.I., Morsi M., Albalawi H., Alamri O.A. (2022). Study of lead-free double perovskites halides Cs_2_TiCl_6_, and Cs_2_TiBr_6_ for optoelectronics, and thermoelectric applications. Mater. Sci. Semicond. Process..

[40] Aslam S., Farooqi A.S., Rahman M.Y.A., Samsuri S.A.M. (2022). Titanium-Based Vacancy-Ordered Double Halide Family in Perovskite Solar Cells. Phys. Status Solidi A.

[41] Wu S., Li W., Hu J., Gao P. (2020). Antimony doped lead-free double perovskites (Cs_2_NaBi_1−x_Sb_x_Cl_6_) with enhanced light absorption and tunable emission. J. Mater. Chem. C.

[42] Schade L., Wright A.D., Johnson R.D., Dollmann M., Wenger B., Nayak P.K., Prabhakaran D., Herz L.M., Nicholas R., Snaith H.J. (2018). Structural and Optical Properties of Cs_2_AgBiBr_6_ Double Perovskite. ACS Energy Lett..

[43] Slavney A.H., Hu T., Lindenberg A.M., Karunadasa H.I. (2016). A Bismuth-Halide Double Perovskite with Long Carrier Recombination Lifetime for Photovoltaic Applications. J. Am. Chem. Soc..

[44] Yang B., Chen J., Yang S., Hong F., Sun L., Han P., Pullerits T., Deng W., Han K. (2018). Lead-Free Silver-Bismuth Halide Double Perovskite Nanocrystals. Angew. Chem. Int. Ed..

[45] Luo R., Zhang S., Zhao S., Li J., Kang F., Yu K., Wei G. (2020). Ultrasmall Blueshift of Near-Infrared Fluorescence in Phase-Stable Cs_2_SnI_6_ Thin Films. Phys. Rev. Appl..

[46] Liu S.-Y., Sun M., Zhang S., Liu S., Li D.-J., Niu Z., Li Y., Wang S. (2021). First-principles study of thermodynamic miscibility, structures, and optical properties of Cs_2_Sn(X_1−x_Y_x_)_6_ (X,Y = I, Br, Cl) lead-free perovskite solar cells. Appl. Phys. Lett..

[47] Huang J., Chang T., Zeng R., Yan J., Wei Q., Zhou W., Cao S., Zou B. (2021). Controlled Structural Transformation in Sb-Doped Indium Halides A_3_InCl_6_ and A_2_InCl_5_∙H_2_O Yields Reversible Green-to-Yellow Emission Switch. Adv. Opt. Mater..

[48] Wei Q., Chang T., Zeng R., Cao S., Zhao J., Han X., Wang L., Zou B. (2021). Self-Trapped Exciton Emission in a Zero-Dimensional (TMA)_2_SbCl_5_.DMF Single Crystal and Molecular Dynamics Simulation of Structural Stability. J. Phys. Chem. Lett..

[49] Chang T., Wei Q., Wang Z., Gao Y., Lian B., Zhu X., Cao S., Zhao J., Zou B., Zeng R. (2022). Phase-Selective Solution Synthesis of Cd-Based Perovskite Derivatives and Their Structure/Emission Modulation. J. Phys. Chem. Lett..

[50] Su B., Li M., Song E., Xia Z. (2021). Sb^3+^-Doping in Cesium Zinc Halides Single Crystals Enabling High-Efficiency Near-Infrared Emission. Adv. Funct. Mater..

[51] Khalfin S., Bekenstein Y. (2019). Advances in lead-free double perovskite nanocrystals, engineering band-gaps and enhancing stability through composition tunability. Nanoscale.

[52] Igbari F., Wang Z.K., Liao L.S. (2019). Progress of Lead-Free Halide Double Perovskites. Adv. Energy Mater..

[53] Lin J., Liu K., Ruan H., Sun N., Chen X., Zhao J., Guo Z., Liu Q., Yuan W. (2022). Zero-Dimensional Lead-Free Halide with Indirect Optical Gap and Enhanced Photoluminescence by Sb Doping. J. Phys. Chem. Lett..

[54] Gautier R., Clerac R., Paris M., Massuyeau F. (2020). Role of specific distorted metal complexes in exciton self-trapping for hybrid metal halides. Chem. Commun..

[55] Zhu D., Zaffalon M.L., Pinchetti V., Brescia R., Moro F., Fasoli M., Fanciulli M., Tang A., Infante I., De Trizio L. (2020). Bright Blue Emitting Cu-Doped Cs_2_ZnCl_4_ Colloidal Nanocrystals. Chem. Mater..

[56] Tan Z., Chu Y., Chen J., Li J., Ji G., Niu G., Gao L., Xiao Z., Tang J. (2020). Lead-Free Perovskite Variant Solid Solutions Cs_2_Sn_1-x_Te_x_Cl_6_: Bright Luminescence and High Anti-Water Stability. Adv. Mater..

[57] Li M., Zhou J., Molokeev M.S., Jiang X., Lin Z., Zhao J., Xia Z. (2019). Lead-Free Hybrid Metal Halides with a Green-Emissive [MnBr_4_] Unit as a Selective Turn-On Fluorescent Sensor for Acetone. Inorg. Chem..

[58] Liu Z., Li Y., Guan X., Mi Y., Al-Hussain A., Ha S.T., Chiu M.H., Ma C., Amer M.R., Li L.J. (2019). One-Step Vapor-Phase Synthesis and Quantum-Confined Exciton in Single-Crystal Platelets of Hybrid Halide Perovskites. J. Phys. Chem. Lett..

[59] Xing J., Liu X.F., Zhang Q., Ha S.T., Yuan Y.W., Shen C., Sum T.C., Xiong Q. (2015). Vapor Phase Synthesis of Organometal Halide Perovskite Nanowires for Tunable Room-Temperature Nanolasers. Nano Lett..

[60] Protesescu L., Yakunin S., Bodnarchuk M.I., Krieg F., Caputo R., Hendon C.H., Yang R.X., Walsh A., Kovalenko M.V. (2015). Nanocrystals of Cesium Lead Halide Perovskites (CsPbX_3_, X = Cl, Br, and I): Novel Optoelectronic Materials Showing Bright Emission with Wide Color Gamut. Nano Lett..

[61] Park J., Joo J., Kwon S.G., Jang Y., Hyeon T. (2007). Synthesis of monodisperse spherical nanocrystals. Angew. Chem. Int. Ed..

[62] Yang Y., Han A., Hao S., Li X., Luo X., Fang G., Liu J., Wang S. (2020). Fluorescent methylammonium lead halide perovskite quantum dots as a sensing material for the detection of polar organochlorine pesticide residues. Analyst.

[63] Zhang J., Hodes G., Jin Z., Liu S.F. (2019). All-Inorganic CsPbX_3_ Perovskite Solar Cells: Progress and Prospects. Angew. Chem. Int. Ed..

[64] Chen D., Yuan S., Chen X., Li J., Mao Q., Li X., Zhong J. (2018). CsPbX_3_ (X = Br, I) perovskite quantum dot embedded low-melting phosphosilicate glasses: Controllable crystallization, thermal stability and tunable emissions. J. Mater. Chem. C.

[65] Zhang B., Pinchetti V., Zito J., Ray A., Melcherts A.E.M., Ghini M., Pianetti A., Infante I., Brovelli S., De Trizio L. (2021). Isolated [SbCl_6_]^3–^ Octahedra Are the Only Active Emitters in Rb_7_Sb_3_Cl_16_ Nanocrystals. ACS Energy Lett..

[66] Cai T., Shi W., Hwang S., Kobbekaduwa K., Nagaoka Y., Yang H., Hills-Kimball K., Zhu H., Wang J., Wang Z. (2020). Lead-Free Cs_4_CuSb_2_Cl_12_ Layered Double Perovskite Nanocrystals. J. Am. Chem. Soc..

[67] Lv K., Qi S., Liu G., Lou Y., Chen J., Zhao Y. (2019). Lead-free silver-antimony halide double perovskite quantum dots with superior blue photoluminescence. Chem. Commun..

[68] Yang H., Guo Y., Liu G., Song R., Chen J., Lou Y., Zhao Y. (2022). Near UV luminescent Cs_2_NaBi_0.75_Sb_0.25_Cl_6_ perovskite colloidal nanocrystals with high stability. Chin. Chem. Lett..

[69] Singh A., Chiu N.C., Boopathi K.M., Lu Y.J., Mohapatra A., Li G., Chen Y.F., Guo T.F., Chu C.W. (2019). Lead-Free Antimony-Based Light-Emitting Diodes through the Vapor-Anion-Exchange Method. ACS. Appl. Mater. Interfaces.

[70] Correa-Baena J.-P., Nienhaus L., Kurchin R.C., Shin S.S., Wieghold S., Putri Hartono N.T., Layurova M., Klein N.D., Poindexter J.R., Polizzotti A. (2018). A-Site Cation in Inorganic A_3_Sb_2_I_9_ Perovskite Influences Structural Dimensionality, Exciton Binding Energy, and Solar Cell Performance. Chem. Mater..

[71] Peng H., Tian Y., Yu Z., Wang X., Ke B., Zhao Y., Dong T., Wang J., Zou B. (2022). (C_16_H_28_N)_2_SbCl_5_: A new lead-free zero-dimensional metal-halide hybrid with bright orange emission. Sci. China Mater..

[72] Jacobs P.W.M. (1991). Alkali halide crystals containing impurity ions with the ns2 ground-state electronic configuration. J. Phys. Chem. Solids.

[73] Oomen E.W.J.L., Smit W.M.A., Blasse G. (1984). Jahn-Teller effect in the Sb^3+^ emission in zircon-structured phosphates. Chem. Phys. Lett..

[74] Hariharan M., Scholes G.D. (2022). Virtual Issue on Triplet Excitons. J. Phys. Chem. Lett..

[75] Peng H., Tian Y., Wang X., Huang T., Xiao Y., Dong T., Hu J., Wang J., Zou B. (2021). Bulk assembly of a 0D organic antimony chloride hybrid with highly efficient orange dual emission by self-trapped states. J. Mater. Chem. C.

[76] Luo J.B., Wei J.H., Zhang Z.Z., Kuang D.B. (2022). Water-Molecule-Induced Emission Transformation of Zero-Dimension Antimony-Based Metal Halide. Inorg. Chem..

[77] Jin J., Peng Y., Xu Y., Han K., Zhang A., Yang X.-B., Xia Z. (2022). Bright Green Emission from Self-Trapped Excitons Triggered by Sb^3+^ Doping in Rb_4_CdCl_6_. Chem. Mater..

[78] Li D.-Y., Song J.-H., Xu Z.-Y., Gao Y.-J., Yin X., Hou Y.-H., Feng L.-J., Yue C.-Y., Fei H., Lei X.-W. (2022). Reversible Triple-Mode Switching in Photoluminescence from 0D Hybrid Antimony Halides. Chem. Mater..

[79] Meng X., Wei Q., Lin W., Huang T., Ge S., Yu Z., Zou B. (2022). Efficient Yellow Self-Trapped Exciton Emission in Sb^3+^-Doped RbCdCl_3_ Metal Halides. Inorg. Chem..

[80] Zhang F., Chen X., Qi X., Liang W., Wang M., Ma Z., Ji X., Yang D., Jia M., Wu D. (2022). Regulating the Singlet and Triplet Emission of Sb^3+^ Ions to Achieve Single-Component White-Light Emitter with Record High Color-Rendering Index and Stability. Nano Lett..

[81] Locardi F., Samoli M., Martinelli A., Erdem O., Magalhaes D.V., Bals S., Hens Z. (2021). Cyan Emission in Two-Dimensional Colloidal Cs_2_CdCl_4_:Sb^3+^ Ruddlesden-Popper Phase Nanoplatelets. ACS Nano.

[82] Zhou C., Lin H., He Q., Xu L., Worku M., Chaaban M., Lee S., Shi X., Du M.-H., Ma B. (2019). Low dimensional metal halide perovskites and hybrids. Mater. Sci. Eng. R Rep..

[83] Mao L., Ke W., Pedesseau L., Wu Y., Katan C., Even J., Wasielewski M.R., Stoumpos C.C., Kanatzidis M.G. (2018). Hybrid Dion-Jacobson 2D Lead Iodide Perovskites. J. Am. Chem. Soc..

[84] Lassoued M.S., Soltan W.B., Abdelbaky M.S.M., Ammar S., Gadri A., Salah A.B., García-Granda S. (2017). Structural, vibrational and optical properties of a new self assembled organic–inorganic crystal (C_4_H_7_N_2_) [CdCl_3_(H_2_O)]. J. Mater. Sci: Mater. Electron..

[85] Majher J.D., Gray M.B., Liu T., Holzapfel N.P., Woodward P.M. (2020). Rb_3_InCl_6_: A Monoclinic Double Perovskite Derivative with Bright Sb^3+^-Activated Photoluminescence. Inorg. Chem..

[86] Wu J., Li X., Lian X., Su B., Pang J., Li M.D., Xia Z., Zhang J.Z., Luo B., Huang X.C. (2021). Ultrafast Study of Exciton Transfer in Sb(III)-Doped Two-Dimensional [NH_3_(CH_2_)_4_NH_3_]CdBr_4_ Perovskite. ACS Nano.

[87] McCall K.M., Morad V., Benin B.M., Kovalenko M.V. (2020). Efficient Lone-Pair-Driven Luminescence: Structure-Property Relationships in Emissive 5s^2^ Metal Halides. ACS Mater. Lett..

[88] Peng H., He X., Wei Q., Tian Y., Lin W., Yao S., Zou B. (2022). Realizing High-Efficiency Yellow Emission of Organic Antimony Halides via Rational Structural Design. ACS. Appl. Mater. Interfaces.

[89] Su B., Geng S., Xiao Z., Xia Z. (2022). Highly Distorted Antimony(III) Chloride [Sb_2_Cl_8_]^2−^ Dimers for Near-Infrared Luminescence up to 1070 nm. Angew. Chem. Int. Ed..

[90] McCall K.M., Stoumpos C.C., Kostina S.S., Kanatzidis M.G., Wessels B.W. (2017). Strong Electron–Phonon Coupling and Self-Trapped Excitons in the Defect Halide Perovskites A_3_M_2_I_9_ (A = Cs, Rb; M = Bi, Sb). Chem. Mater..

[91] Tan Z., Hu M., Niu G., Hu Q., Li J., Leng M., Gao L., Tang J. (2019). Inorganic antimony halide hybrids with broad yellow emissions. Sci. Bull..

[92] Huang T., Li K., Lei J., Niu Q., Peng H., Zou B. Origin of singlet self-trapped exciton and enhancement of photoluminescence quantum yield of organic-inorganic hybrid antimony(III) chlorides with the [SbCl_5_]^2−^ units. Nano Res..

[93] Ma Z., Shi Z., Yang D., Zhang F., Li S., Wang L., Wu D., Zhang Y., Na G., Zhang L. (2019). Electrically-Driven Violet Light-Emitting Devices Based on Highly Stable Lead-Free Perovskite Cs_3_Sb_2_Br_9_ Quantum Dots. ACS Energy Lett..

[94] Wei Q., Meng X., Lin W., Ge S., Han X., Chen L., Zeng R., Zou B. (2022). Green Triplet Self-Trapped Exciton Emission in Layered Rb_3_Cd_2_Cl_7_:Sb^3+^ Perovskite: Comparison with RbCdCl_3_:Sb^3+^. J. Phys. Chem. Lett..

[95] Lin W., Wei Q., Huang T., Meng X., Tian Y., Peng H., Zou B. (2023). Antimony doped tin(iv) hybrid metal halides with high-efficiency tunable emission, WLED and information encryption. J. Mater. Chem. C.

[96] Li C., Luo Z., Liu Y., Wei Y., He X., Chen Z., Zhang L., Chen Y., Wang W., Liu Y. (2022). Self-Trapped Exciton Emission with High Thermal Stability in Antimony-Doped Hybrid Manganese Chloride. Adv. Opt. Mater..

[97] Wu Y., Li J., Zheng D., Xia X., Yang S., Yang Y., Bai T., Wang X., Chen J., Yang B. (2022). Ultrasensitive Optical Thermometry via Inhibiting the Energy Transfer in Zero-Dimensional Lead-Free Metal Halide Single Crystals. J. Phys. Chem. Lett..

[98] Zhou J., Rong X., Molokeev M.S., Zhang X., Xia Z. (2018). Exploring the transposition effects on the electronic and optical properties of Cs_2_AgSbCl_6_ via a combined computational-experimental approach. J. Mater. Chem. A.

[99] Noculak A., Morad V., McCall K.M., Yakunin S., Shynkarenko Y., Worle M., Kovalenko M.V. (2020). Bright Blue and Green Luminescence of Sb(III) in Double Perovskite Cs_2_MInCl_6_ (M = Na, K) Matrices. Chem. Mater..

[100] Gray M.B., Hariyani S., Strom T.A., Majher J.D., Brgoch J., Woodward P.M. (2020). High-efficiency blue photoluminescence in the Cs_2_NaInCl_6_:Sb^3+^ double perovskite phosphor. J. Mater. Chem. C.

[101] Chang T., Wang H., Gao Y., Cao S., Zhao J., Zou B., Zeng R. (2022). Component Engineering to Tailor the Structure and Optical Properties of Sb-Doped Indium-Based Halides. Inorg. Chem..

[102] Biswas A., Bakthavatsalam R., Mali B.P., Bahadur V., Biswas C., Raavi S.S.K., Gonnade R.G., Kundu J. (2021). The metal halide structure and the extent of distortion control the photo-physical properties of luminescent zero dimensional organic-antimony(iii) halide hybrids. J. Mater. Chem. C.

[103] Zhang Z.Z., Jin J.C., Gong L.K., Lin Y.P., Du K.Z., Huang X.Y. (2021). Co-luminescence in a zero-dimensional organic-inorganic hybrid antimony halide with multiple coordination units. Dalton Trans..

[104] Shi C.-M., Li J.-L., Xu L.-J., Wu Y., Xuan H.-L., Wang J.-Y., Chen Z.-N. (2022). Methanol-induced luminescence vapochromism based on a Sb^3+^-doped organic indium halide hybrid. Sci. China Mater..

[105] McCall K.M., Benin B.M., Wörle M., Vonderach T., Günther D., Kovalenko M.V. (2020). Expanding the 0D Rb_7_M_3_X_16_ (M = Sb, Bi; X = Br, I) Family: Dual-Band Luminescence in Rb_7_Sb_3_Br_16_. Helv. Chim. Acta.

[106] Song G., Li M., Zhang S., Wang N., Gong P., Xia Z., Lin Z. (2020). Enhancing Photoluminescence Quantum Yield in 0D Metal Halides by Introducing Water Molecules. Adv. Funct. Mater..

[107] Zhou C., Worku M., Neu J., Lin H., Tian Y., Lee S., Zhou Y., Han D., Chen S., Hao A. (2018). Facile Preparation of Light Emitting Organic Metal Halide Crystals with Near-Unity Quantum Efficiency. Chem. Mater..

[108] Hu Y., Wei Q., Xing K., Li X., Yu P., Chen L., Zhong X., Zou B. (2022). Highly efficient and stable red-emitting Sb-doped Indium-based perovskites via anionic component engineering. Mater. Res. Bull..

[109] Qiao J., Zhou G., Zhou Y., Zhang Q., Xia Z. (2019). Divalent europium-doped near-infrared-emitting phosphor for light-emitting diodes. Nat. Commun..

[110] Manley M. (2014). Near-infrared spectroscopy and hyperspectral imaging: Non-destructive analysis of biological materials. Chem. Soc. Rev..

[111] Xie R.-J. (2020). Light-emitting diodes: Brighter NIR-emitting phosphor making light sources smarter. Light-Sci. Appl..

[112] Mao L., Wu Y., Stoumpos C.C., Wasielewski M.R., Kanatzidis M.G. (2017). White-Light Emission and Structural Distortion in New Corrugated Two-Dimensional Lead Bromide Perovskites. J. Am. Chem. Soc..

[113] Peng H., Xiao Y., Tian Y., Wang X., Huang T., Dong T., Zhao Y., Wang J., Zou B. (2021). Dual self-trapped exciton emission of (TBA)_2_Cu_2_I_4_: Optical properties and high anti-water stability. J. Mater. Chem. C.

[114] Chang T., Wei Q., Zeng R., Cao S., Zhao J., Zou B. (2021). Efficient Energy Transfer in Te^4+^-Doped Cs_2_ZrCl_6_ Vacancy-Ordered Perovskites and Ultrahigh Moisture Stability via A-Site Rb-Alloying Strategy. J. Phys. Chem. Lett..

[115] Feng S., Ma Y., Wang S., Gao S., Huang Q., Zhen H., Yan D., Ling Q., Lin Z. (2022). Light/Force-Sensitive 0D Lead-Free Perovskites: From Highly Efficient Blue Afterglow to White Phosphorescence with Near-Unity Quantum Efficiency. Angew. Chem. Int. Ed..

[116] Lin H., Wei Q., Ke B., Lin W., Zhao H., Zou B. (2023). Excitation-Wavelength-Dependent Emission Behavior in (NH_4_)_2_SnCl_6_ via Sb^3+^ Dopant. J. Phys. Chem. Lett..

[117] Li J.L., Sang Y.F., Xu L.J., Lu H.Y., Wang J.Y., Chen Z.N. (2022). Highly Efficient Light-Emitting Diodes Based on an Organic Antimony(III) Halide Hybrid. Angew. Chem. Int. Ed..

[118] Qi Z., Chen Y., Gao H., Zhang F.-Q., Li S.-L., Zhang X.-M. (2021). Two SbX_5_-based isostructural polar 1D hybrid antimony halides with tunable broadband emission, nonlinear optics, and semiconductor properties. Sci. China Chem..

[119] Sun C., Deng Z., Li Z., Chen Z., Zhang X., Chen J., Lu H., Canepa P., Chen R., Mao L. (2023). Achieving Near-unity Photoluminescence Quantum Yields in Organic-Inorganic Hybrid Antimony (III) Chlorides with the [SbCl_5_] Geometry. Angew. Chem. Int. Ed..

[120] Morad V., Yakunin S., Kovalenko M.V. (2020). Supramolecular Approach for Fine-Tuning of the Bright Luminescence from Zero-Dimensional Antimony(III) Halides. ACS Mater. Lett..

[121] Peng H., Wang X., Tian Y., Zou B., Yang F., Huang T., Peng C., Yao S., Yu Z., Yao Q. (2021). Highly Efficient Cool-White Photoluminescence of (Gua_)3_Cu_2_I_5_ Single Crystals: Formation and Optical Properties. ACS Appl. Mater. Interfaces.

[122] Lu P., Lu M., Wang H., Sui N., Shi Z., Yu W.W., Zhang Y. (2019). Metal halide perovskite nanocrystals and their applications in optoelectronic devices. InfoMat.

[123] Brites C.D.S., Lima P.P., Silva N.J.O., Millán A., Amaral V.S., Palacio F., Carlos L.D. (2011). Lanthanide-based luminescent molecular thermometers. New J. Chem..

[124] Yin J., Zhang G., Peng C., Fei H. (2019). An ultrastable metal-organic material emits efficient and broadband bluish white-light emission for luminescent thermometers. Chem. Commun..

[125] Lu X., Peng H., Wei Q., Lin W., Tian Y., Li T., Zhou S., Zhao J., Zou B. (2023). Bulk assemblies of organic antimony chloride with multiple reversible photoluminescence switching for anti-counterfeiting and information encryption. Mater. Today Phys..

[126] Ge S., Peng H., Wei Q., Shen X., Huang W., Liang W., Zhao J., Zou B. (2023). Realizing Color-Tunable and Time-Dependent Ultralong Afterglow Emission in Antimony-Doped CsCdCl_3_ Metal Halide for Advanced Anti-Counterfeiting and Information Encryption. Adv. Opt. Mater..

[127] Zhu D., Zaffalon M.L., Zito J., Cova F., Meinardi F., De Trizio L., Infante I., Brovelli S., Manna L. (2021). Sb-Doped Metal Halide Nanocrystals: A 0D versus 3D Comparison. ACS Energy Lett..

[128] Mo Q., Qian Q., Shi Y., Cai W., Zhao S., Zang Z. (2022). High Quantum Efficiency of Stable Sb-Based Perovskite-Like Halides toward White Light Emission and Flexible X-Ray Imaging. Adv. Opt. Mater..

